# Strategies for improving ED-related outcomes of older adults who seek care in emergency departments: a systematic review

**DOI:** 10.1186/s12245-024-00584-7

**Published:** 2024-02-01

**Authors:** Ally Memedovich, Benedicta Asante, Maha Khan, Nkiruka Eze, Brian R. Holroyd, Eddy Lang, Sherri Kashuba, Fiona Clement

**Affiliations:** 1 Department of Community Health Sciences, University of Calgary, Calgary, AB Canada; 2 Department of Emergency Medicine, University of Alberta, Edmonton, AB Canada; 3 Department of Emergency Medicine, University of Calgary, Calgary, AB Canada; 4 Emergency Strategic Clinical Network, Alberta Health Services, Edmonton, AB Canada; 5 O’Brien Institute of Public Health, University of Calgary, Calgary, AB Canada

**Keywords:** Emergency department, Frequent use, Older adults, Systematic review

## Abstract

**Background:**

Despite constituting 14% of the general population, older adults make up almost a quarter of all emergency department (ED) visits. These visits often do not adequately address patient needs, with nearly 80% of older patients discharged from the ED carrying at least one unattended health concern. Many interventions have been implemented and tested in the ED to care for older adults, which have not been recently synthesized.

**Methods:**

A systematic review was conducted to identify interventions initiated in the ED to address the needs of older adults. Embase, MEDLINE, CINAHL, Cochrane CENTRAL, the Cochrane Database of Systematic Reviews, and grey literature were searched from January 2013 to January 18, 2023. Comparative studies assessing interventions for older adults in the ED were included. The quality of controlled trials was assessed with the Cochrane risk-of-bias tool for randomized trials, and the quality of observational studies was assessed with the risk of bias in non-randomized studies of interventions tool. Due to heterogeneity, meta-analysis was not possible.

**Results:**

Sixteen studies were included, assessing 12 different types of interventions. Overall study quality was low to moderate: 10 studies had a high risk of bias, 5 had a moderate risk of bias, and only 1 had a low risk of bias. Follow-up telephone calls, referrals, geriatric assessment, pharmacist-led interventions, physical therapy services, care plans, education, case management, home visits, care transition interventions, a geriatric ED, and care coordination were assessed, many of which were combined to create multi-faceted interventions. Care coordination with additional support and early assessment and intervention were the only two interventions that consistently reported improved outcomes. Most studies did not report significant improvements in ED revisits, hospitalization, time spent in the ED, costs, or outpatient utilization. Two studies reported on patient perspectives.

**Conclusion:**

Few interventions demonstrate promise in reducing ED revisits for older adults, and this review identified significant gaps in understanding other outcomes, patient perspectives, and the effectiveness in addressing underlying health needs. This could suggest, therefore, that most revisits in this population are unavoidable manifestations of frailty and disease trajectory. Efforts to improve older patients’ needs should focus on interventions initiated outside the ED.

**Supplementary Information:**

The online version contains supplementary material available at 10.1186/s12245-024-00584-7.

## Background

Older adults—adults aged 65 or older—contribute to almost a quarter of all visits to the emergency department (ED), despite constituting just 14% of the general population in high-income countries [1–5]. This trend is projected to persist, with an anticipated 30% increase in ED utilization as patients age [4]. However, even after receiving care in the ED, the needs of older adults often remain unaddressed: nearly 80% of older adults discharged from the ED carry at least one unattended health concern [4]. Further, within 6 months of discharge from their initial ED visit, almost 44% of older adults revisit the ED at least once, and around 7.5% return three or more times [4]. While reattendance may be the result of disease progression or overall frailty, given the large proportion of patients with unattended health concerns, it is also likely that at least some patients return due to their needs being unmet in the ED.

These concerning rates of return visits and unfavourable outcomes following the initial ED visit underscore the need to think differently about the ED model of care to address the complex health needs of older patients [1, 4, 6, 7]. Compared to younger patients, older adults are more likely to have age-related visual, hearing, or cognitive impairments, multiple comorbidities, atypical symptoms or disease states, be on multiple medications, and have more complex psychosocial needs [2, 8, 9]. Given the rapid-care ED model, designed for trauma and acute conditions, which often concentrates solely on the immediate issue, EDs as they are currently structured may be unable to address older patients’ unique, complex health challenges [5, 8, 10–12]. Consequently, the substantial health needs of older adults are likely being left unmet [5].

Community-centred approaches and strategies, such as improving the availability and accessibility of primary care services, extending operating hours—especially during off-peak periods—and implementing primary care interventions like nurse-led walk-in centres designed for low-acuity cases, can be highly effective in reducing unplanned ED visits among older adults. However, these interventions are beyond the scope of ED practitioners to implement [13, 14]. Additionally, not all older adults will be able to access community-based services, and EDs may be the only avenue they have to access care. Therefore, given the mounting strain on ED services and the need for older adults to utilize the ED, there is a pressing need for effective interventions to support older adults and ensure their care needs are being met within the ED.

Existing systematic reviews have explored strategies for ED avoidance for older adults; however, nearly all focus on community-based or system-wide interventions rather than interventions implemented specifically in EDs [3, 5, 13]. Further, a 2019 review on ED-based interventions for older adults reported mixed results, particularly for ED-related outcomes, but only focused on four types of ED-based interventions [9]. A recent, comprehensive review of ED-based interventions specifically for older adults is lacking. The objective of this systematic review was to identify interventions implemented in the ED to improve ED-related outcomes in older adults.

## Methods

### Search strategy

A systematic review following Cochrane best practices guidelines and PRISMA reporting standards was conducted [15, 16]. Embase, MEDLINE, CINAHL, Cochrane CENTRAL, and the Cochrane Database of Systematic Reviews were searched. Given the large volume of studies expected and the desire to provide the most up-to-date evidence, the search was limited to the last 10 years. The search was limited to studies published from 2013 to January 18, 2023.

The strategies utilized a combination of MeSH terms (e.g. “emergency service”, hospital”, “patient readmission”, “evaluation study”) and keywords (e.g. “emergency department”, “hotspot”, “intervention study”) to capture interventions of interest. Vocabulary and syntax were adjusted across the databases. The search was limited to English and French language studies. No other filters were applied. The search strategy was developed by a research librarian, and a peer review of the electronic search strategy (PRESS) was conducted by another research librarian [17]. The full search strategy is available in Additional file 1: Appendix A.

Grey literature searches were conducted through the Canadian Agency for Drug and Technologies in Health Grey Matters database, targeted Google searches, and preprint databases including medRixV and Research Square. Canadian provincial health websites were searched for relevant studies or reports. International agency websites including the National Institute for Health and Care Excellence (UK) and Europe PMC were also searched. Additionally, the reference lists of relevant systematic reviews and included studies were hand-searched to ensure all relevant literature was captured.

Records were downloaded, and duplicates were removed using EndNote version 9.3.3 (Clarivate Analytics).

### Study selection

A calibration exercise was conducted by four reviewers on a sample of the retrieved abstracts. A sample of 100 abstracts was reviewed until 100% agreement was reached among reviewers. After 100% agreement was reached, the remaining abstracts were screened in duplicate by two teams of two independent reviewers. Abstracts proceeded to full-text review if they met the following inclusion criteria: assessed the effectiveness of interventions to reduce ED utilization by older adults, interventions were initiated in the ED, comparative study design, and reported on outcomes including but not limited to ED revisits, ED wait times, hospitalization, use of primary care, and costs (Table 1). Abstracts were excluded if they failed to meet the inclusion criteria above or if they were published in languages other than English or French. Abstracts selected for inclusion by either reviewer proceeded to full-text review. This initial screen was intentionally broad to ensure that all relevant literature was captured.

**Table 1:** Inclusion criteria

**Population**	Older adults (at least 90% of the sample > 65 years of age)
Intervention	Any intervention offered in the setting of the ED with an implied or stated goal to reduce ED use
Comparator	Any comparator including pre-intervention as a historical control
Outcomes	Effectiveness of intervention. Measures include, but are not limited to, reduced ED visits, time spent in ED, ED wait times, clinical outcomes, mortality, hospitalization, healthcare system use, and costs
Study design	Any comparative study design including, but not limited to, RCTs, comparative cohort studies, before and after comparative cohort studies
Languages	English or French
Publication date	After 2013

A similar calibration exercise was conducted by all reviewers on a sample of the retrieved full-text studies. A sample of six full texts was reviewed until 100% agreement was reached. After 100% agreement was reached among reviewers, full-text review was conducted in duplicate by two independent reviewers. Any discrepancies between reviewers were resolved through discussion and consensus. If required, a third reviewer was consulted. Full texts were included if they met the above inclusion criteria.

### Data extraction

For all included studies, year of publication, country, study design, participant characteristics, general intervention, intervention details, healthcare practitioner involved in interventions, and outcomes were extracted by a single reviewer using standardized data extraction forms. A second reviewer verified the extracted data. Discrepancies between reviewers during data extraction were resolved through consensus.

### Quality assessment

The quality of controlled trials was assessed using the revised Cochrane risk-of-bias tool for randomized trials (ROB-2) [18], while the non-randomized studies were assessed with the risk of bias in non-randomized studies of interventions (ROBINS-I) tool [19]. Each controlled trial was assessed using five criteria broadly covering the areas of randomization, deviation from intended intervention, missing outcome data, measurement of outcome, and selection of reporting the result. Each criterion was assigned a rating of “low,” “some,” or “high” concern. The observational studies were assessed based on the following parameters: bias due to confounding, selection bias, bias in classification, bias due to deviations from intended interventions, bias due to missing data, bias in measurement, and reporting bias. Each criterion was also assigned a rating of “low,” “moderate”, or “serious” risk of bias. Quality assessment was completed by one reviewer and checked by another independent reviewer. Discrepancies were resolved through discussion. Studies were not excluded based on quality assessment.

### Data analysis

Given the broad range of interventions and outcomes allowed by the inclusion criteria, significant heterogeneity of studies was expected. Therefore, a narrative approach to synthesis was adopted a priori. It was anticipated that meta-analysis would not be possible. The types of interventions used, the outcomes reported, the effectiveness, overall trends, and any gaps in the literature were assessed.

## Results

### Overall findings

**Fig. 1:**
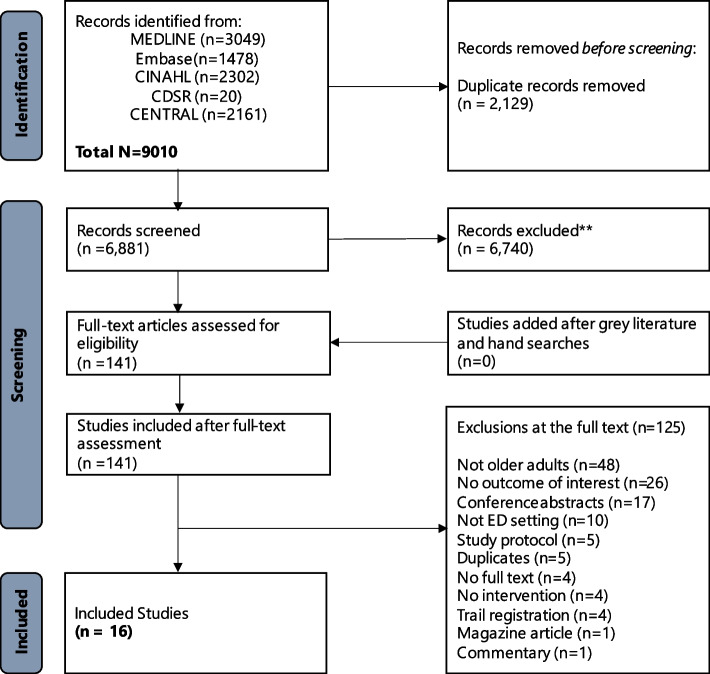
PRIMSA diagram

The search strategy yielded 6881 unique citations, 6740 of which were excluded after abstract review. One-hundred and 41 studies proceeded to full-text review. Studies were excluded for the following reasons: not older adults (*n* = 48), no outcome of interest (*n* = 26), conference abstract (*n* = 17), not ED setting (*n* = 10), study protocol (*n* = 5), duplicates (*n* = 5), no full text (*n* = 4), no intervention (*n* = 4), trial registration (*n* = 4), magazine article (*n* = 1), and commentary (*n* = 1) (Fig. 1).

**Fig. 2:**
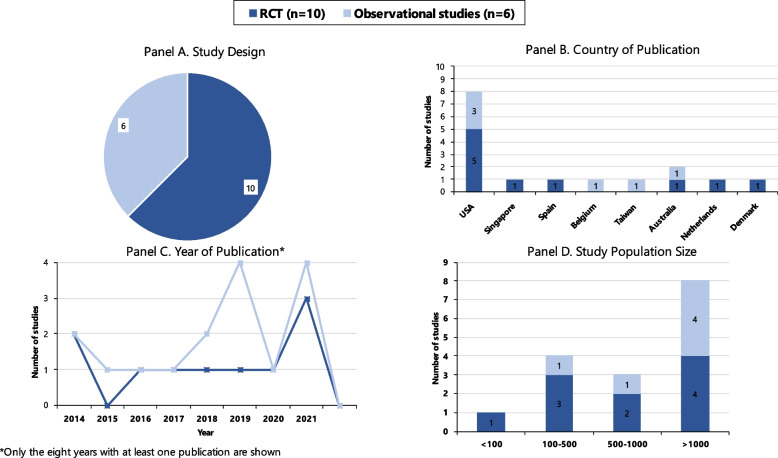
Characteristics of included studies

Ten controlled trials and six observational studies assessed interventions for older adults (Table 2). Most studies (*n* = 8) were conducted in the USA [20–26], two were from Australia [27, 28], and one each were from Belgium [29], Denmark [30], Singapore [31], Spain [32], the Netherlands [33], and Taiwan [34] and were published from 2014 to 2021 with no particular concentration (Fig. 2).

**Table 2:** Detailed information on included studies

Study information		Patient characteristics	ED visits	Other outcomes
Follow-up telephone call				
Biese et al. (2014) [20], USA Trial no.: NCT01207180 Funder: Duke Endowment and the Community Connection for Seniors Industry sponsored: no	Study design: RCT Intervention type: follow-up telephone call Comparator: TAU and placebo	*N*: intervention = 39; control = 46; placebo = 35 Age (mean): 75 years % female: intervention = 59%; control = 61%; placebo = 60%	22% of intervention patients revisited ED compared to 33% of placebo and 27% of control (*p* = 0.41)	Costs: intervention had a 70% chance of being cost saving and a 3% chance of being cost-effective and 27% chance of resulting in more return visits at higher total costs
Biese et al. (2018) [21], USA Trial no.: NCT01893931z Funder: Duke Endowment, the Kenan Family Foundation, and Mr. John A. McNeill, Jr Industry sponsored: no	Study design: RCT Intervention type: follow-up telephone call Comparator: control group	*N*: intervention = 974; control = 975 Age (mean): intervention = 74.2 years; control = 73.9 years % female: intervention = 59.1%; control = 61.1%	Rate of return to ED, hospitalization, or death: 15.5% (95% *CI*: 13.2–17.8) in intervention; 15.2% (95% *CI*: 12.9–17.5) in control, *p* = 0.86 Return to ED: 12.2% (95% *CI*: 10.1–14.3) in intervention; 12.5% (95% *CI*: 10.4–14.6) in control	Hospitalization within 30 days: 9% (95% *CI*: 7.2–10.8) in intervention; 7.4% (95% *CI*: 5.8–9.0) in control Outpatient use, seeing a PCP within 30 days: 80.8% in both groups Stratifying by age did not find a benefit
van Loon-van Gaalen et al. (2021) [11, 33], Netherlands Trial no.: trial NL6598 Funder: Jacobus Foundation Industry sponsored: no	Study design: RCT Intervention type: follow-up telephone call Comparator: control group	*N*: intervention = 1516; control = 1659 Age (median): intervention = median 78 years; control = median 78 years % female: intervention = 58%; control = 58%	Unplanned hospital admission and/or ED revisit within 30 days: 16% of intervention; 14% of control, *OR* 1.16, 95% *CI*: 0.96–1.42; separate rates were not significant	Stratifying by age: patients aged < 78 years: intervention patients had more unplanned hospital admissions and/or ED revisits than control pts. (18% vs 14%, *OR* 1.33, 95% *CI*: 1.01–1.75)
Comprehensive geriatric assessment + other				
Foo et al. (2014) [31], Singapore Trial no.: National Healthcare Group (NHG) Domain-Specific Review Board (DSRB) C/09/023 Funder: Ministry of Health’s Healthcare Quality Improvement and Innovation (HQI2) Fund Industry sponsored: no	Study design: RCT Intervention type: geriatric assessment, referral Comparator: TAU	*N*: intervention = 234; control = 425 Age (median): intervention = 77 years; control = 77 years % female: intervention = 53.6%; control = 56.2%	ED reattendance, 3 months: 37.2% in control; 36.8% in intervention, *p* = 0.97, adjusted *OR* = 0.91, 95% *CI*: 0.67–1.24 ED reattendance, 6 months: 50.8% in control; 47.9% in intervention, *p* = 0.84, *OR*: 0.82, 95% *CI*: 0.61–1.11 ED reattendance, 9 months: 59.8% in control; 54.6% in intervention, *p* = 0.19, *OR*: 0.74, 95% *CI*: 0.55–1.01 ED reattendance, 12 months: 66.0% in control; 61.1% in intervention, *p* = 0.19, *OR*: 0.75, 95% *CI*: 0.55–1.03	Hospitalization, 3 months: 28.8% of control; 27.9% of intervention, *p* = 0.84, adjusted *OR*: 0.88, 95% *CI*: 0.63–1.22 Hospitalization, 6 months: 40.4% in control; 38.2% in intervention, *p* = 0.60, *OR*: 0.84, 95% *CI*: 0.62–1.14 Hospitalization, 9 months: 48.2% in control; 43.9% in intervention; *p* = 0.28, *OR*: 0.76, 95% *CI*: 0.56–1.03 Hospitalization, 12 months: 53.8% in control; 49.6% in intervention, *p* = 0.30, *OR*: 0.77, 95% *CI*: 0.57–1.04
Heeren et al. (2019) [29], Belgium Trial no.: ISRCTN91449949 Funder: the Flemish government agency for Innovation by Science and Technology Industry sponsored: no	Study design: observational study Intervention type: geriatric assessment tool, care plan, case-manager follow-up, referral to geriatric clinic Comparator: cohort collected prior to intervention period	*N*: intervention = 886; control = 794 Age (Q2): intervention = 81 years; control = 80 years % female: intervention = 52.9%; control = 54.9%	Unplanned ED readmission, 30 days: 12.1% in control; 13.1% in intervention, *p* = 0.21 Unplanned ED readmission, 90 days: 22.1% in control; 23.9% in intervention, *p* = 0.11 Median time to unplanned ED readmission within 90 days: 25.1 days (min 0.3, max 88.3) for control; 27.6 (min 0.2, max 88.0) days in intervention, *p* = 0.66	Median ED LOS: 19.1 h in control; 12.7 h in intervention, *p* < 0.001 Hospitalization: 70.0% in intervention; 67.0% in control, *p* = 0.003 Median hospital LOS: 8.7 days in control; 8.6 days in intervention, *p* = 0.15
Lin et al. (2021) [34], Taiwan Trial no.: IRB no. CE18256 Funder: Veterans Affairs Council, Taiwan Industry sponsored: no	Study design: observational study Intervention type: comprehensive geriatric assessment, case management Comparator: TAU	*N*: intervention = 236; control = 122 Age (median): intervention = 82 years; control = 82 years % female: intervention = 50.4%; control = 69.7%	ED revisits within three months: 35.3% pre-intervention; 28.4% post-intervention Not statistically significant	Hospitalization: decreased by 27% (50.8% pre-intervention, 23.1% post-intervention) Male gender associated with decreased ORs of admission following index ED visit
Pedersen et al. (2016) [30], Denmark Trial no.: none reported Funder: none reported Industry sponsored: not reported	Study design: RCT Intervention type: geriatric assessment, home visit by geriatrician and nurse Comparator: TAU	*N*: intervention = 693; control = 637 Age (mean): intervention = 86.4 years; control = 86.4 years % female: intervention = 60%; control = 64%	None reported	Discharges: 56% of intervention patients were discharged directly from the ED compared to 46% of control, *p* = 0.01 Hospitalization: 12% in intervention; 23% in control, *p* < 0.001 Of those admitted, LOS for intervention patients (median = 2 days) was significantly shorter than for control (median = 3 days), *p* = 0.03
Pharmacist-lead intervention				
Santolaya-Perrin et al. (2019) [32], Spain Trial no.: none reported Funder: RED-FASTER of SEFH (Sociedad Española de Farmacia Hospitalaria) Industry sponsored: no	Study design: RCT Intervention type: medication review programme Comparator: TAU	*N*: intervention = 323; control = 342 Age (mean): intervention = 78.99 years; control = 78.2 years % female: intervention = 51.6%; control = 53.5%	Emergency visits and hospital admissions: no significant differences between groups RR, 95% *CI*: 0.857, 0.652–1.126 for 3 months RR, 95% *CI*: 0.917, 0.715–1.176 for 6 months RR, 95% CI: 0.954, 0.766–1.187 for 12 months	None reported
Shaw et al. (2016) [26], USA Trial name: EMBRACE ED Funder: Kaiser Permanente Colorado Industry sponsored: yes	Study design: observational study Intervention type: pharmacist intervention Comparator: non-clinical pharmacist + elder ED, and non-elder ED + clinical pharmacist	*N*: intervention = 4103 patients in total; 342 treated by EMBRACE clinical pharmacy specialist; control = 530 treated with non-clinical pharmacy specialist but in EMBRACE; 3231 non-EMBRACE Age (mean): 77 years overall % female: 58% overall	Patients in CPS + EMBRACE group more likely to experience a 30-day return visit than those in non-EMBRACE group (unadjusted *OR* 1.42, 95% *CI*: 1.09–1.85), and a 90-day return visit than those in the non-EMBRACE group (unadjusted *OR* 1.34, 95% *CI*: 1.06–1.69) 30-day ED return visit: 24% in intervention, 18.2% in non-EMBRACE, 18.9% in non-CPS 90-day ED return visit: 36.3% in intervention, 29.8% in non-EMBRACE, 30.6% in non-CPS	Admitted from index ED visit: 42.4% in intervention, 42.1% in non-EMBRACE, 42.5% in non-CPS; no differences
Care transition intervention				
Schumacher et al. (2021) [35], USA Trial no.: NCT02079987 Funder: Patient-Centered Outcomes Research Institute award Industry sponsored: no	Study design: RCT Intervention type: care transition intervention Comparator: TAU	Definition of frequent user: 3 or more visits in prior year N: intervention = 557; control = 544 Age (mean): intervention = 72.4 years; control = 72.8 years % female: intervention = 60%; control = 63%	Post-intervention: 627 return ED visits made within 60 days ED visit: *OR* 1.08 (95% *CI*: 0.83–1.39), not statistically significant	Intervention did not significantly affect hospital-based acute care, but ED return visits were less likely to result in hospitalization Hospital admission at index ED visit: intervention participants had 36% lower odds of hospitalization upon ED return (*OR* 0.64, 95% *CI*: 0.45–0.91) Outpatient visit: *OR* 1.13 (95% *CI*: 0.77–1.67), not statistically significant Reasons for ED return visits: patients were confident they would get needed care in the ED, PCP often encourage patients to seek emergency care Reasons for not visiting outpatient clinics: barriers to timely outpatient care, difficulty scheduling appointments, office-based outpatient visits discouraged if diagnostic tests were required Physician perspective on hospital admissions: ED physicians often hospitalize patients if outpatient follow-up or social support are in question
Early assessment and intervention				
Cassarino et al. (2021) [22], USA Trial no.: NCT03739515 Funder: Health Research Board of Ireland through the Research Collaborative for Quality and Patient Safety Industry sponsored: no	Study design: RCT Intervention type: early assessment and intervention Comparator: TAU	*N*: intervention = 176; control = 177 Age (mean): intervention = 78.6 years; control = 80.6 years % female: intervention = 61.4%; control = 57.1%	30-day ED revisit: 18.8% for intervention; 13.6% for control, *OR* 1.42 (95% *CI*: 0.79–2.55), *p* = 0.23 6-month ED revisit: 31.4% for intervention; 43.3% for control, *OR* = 0.65 (95% *CI*: 0.42–1.02), *p* = 0.06	Time spent in ED, index visit, median (IQR), hours: 6.43 (4.05–14.87) for intervention; 12.1 (6.18–22.14) for control, *p* < 0.001 Hospitalization at index visit: 19.3% for intervention; 55.9% for control, *p* < 0.001 Unscheduled hospital admission at 30 days: 11.9% for intervention; 12.4% for control, *OR* 0.96 (95% *CI*: 0.51–1.84), *p* = 0.92 Unscheduled hospital admission at 6 months: 19.4% for intervention; 33.3% for control, *OR* = 0.52 (95% *CI*: 0.32–0.88), *p* = 0.02 Hospital LOS at index visit, median (IQR), days: 9 (3–13) for intervention; 9 (5–24) for control, *p* = 0.32 Satisfaction at index visit, mean (SD): 25.8 (3.03) for intervention; 24.8 (3.74) for control, *p* = 0.008
Physical therapy				
Lesser et al. (2018) [24], USA Trial no.: none reported Funder: none reported Industry sponsored: not reported	Study design: observational study Intervention type: physical therapy services Comparator: TAU	*N*: intervention = 17,791; control = 173,651 Age (mean): intervention = 82.4 years; control = 80.6 years % female: intervention = 70.4%; control = 66.2%	30-day follow-up, all-cause ED: 20.4% in intervention; 21.7% in control Fall-related ED revisits at 30 days: 1.7% in intervention; 2.6% for control Fall-related ED revisits at 60 days: 2.5% in intervention; 3.6% in control *p* < 0.01 for both 30 and 60 days	None reported
Multi-faceted interventions				
Arendts et al. (2018) [27], Australia Trial no.: ACTRN12612000798864 Funder: State Health Research Advisory Council of Western Australia Industry sponsored: no	Study design: RCT Intervention type: education, follow-up telephone call, referral Comparator: TAU	*N*: intervention = 81; control = 80 Age (mean): intervention = 78 years; control = 78 years % female: intervention = 39%; control = 37%	8% absolute (95% *CI*: 7–20) and 20% relative risk reduction for an intervention patient making an unplanned ED reattendance within 28 days	Hospitalization: no significant difference in 28-day hospitalization rates or hospital bed day usage
Goldberg et al. (2020) [23], USA Trial no.: NCT03360305 Funder: National Institute on Aging and Society for Academic Emergency Medicine Foundation/Emergency Medicine Foundation GEMSSTAR for Emergency Medicine Supplemental Funding Industry sponsored: no	Study design: RCT Intervention type: pharmacist-led medication review; physical therapy consultation Comparator: TAU	*N*: intervention = 55; control = 55 Age (median): intervention = 81.9 years; control = 80.1 years % female: intervention = 67%; control = 67%	Total visits: control = 66; intervention = 30 Adjusted rate of all ED visits: control = 1.54 (95% *CI*: 1.04–2.30); intervention = 0.73 (95% *CI*: 0.45–1.17), *IRR*: 0.47 (95% *CI*: 0.29–0.74)	ED LOS: control = 5.3 h; intervention = 5 h, *p* < 0.94 Hospitalization: control = 34; intervention = 19; adjusted rate: control = 0.77 (95% *CI*: 0.46–1.31); intervention = 0.44 (95% *CI*: 0.24–0.82); *IRR*: 0.57 (95% *CI*: 0.31–1.04)
Liberman et al. (2020) [25], USA Trial name: the GAP-ED project (Geriatric and Palliative Emergency Department) Funder: Fan Fox and Leslie R. Samuels Foundation Industry sponsored: no	Study design: observational study Intervention type: care plan, education, referral Comparator: historical usual-care group	*N*: intervention = 283; control = 283 Age: intervention = 11% 65–75 years, 40% 76–85 years, 44% 86–94 years, 5% 95 + ; control = 11% 65–75 years, 40% 76–85 years, 44% 86–94 years, 5% 95 + % female: intervention = 78%; control = 78%	Average revisits within 30 days: 0.22 in control, 0.20 in intervention, *p* = 0.34 In both groups, over 80% of patients had no revisits	Hospitalization: of those who revisited within 30 days, 40% of intervention were admitted, and 57% of control were admitted, *p* = 0.001
Shrapnel et al. (2019) [28], Australia Trial no.: none reported Funder: the Mater Hospital Brisbane funded one full-time nursing position to implement the MACIAE study; co-author ED is funded by an Australian National Health and Medical Research Council Early Career Fellowship Industry sponsored: no	Study design: observational study Intervention type: care coordination, support Comparator: TAU	*N*: intervention = 391; control = 730 Age (not specified): intervention = 83.1 years; control = 84.8 years % female: not reported	Revisit within 28 days: 4.6% for intervention; 17.8% for control, *p* < 0.001	Admission after ED presentation: 40.6% for intervention; 71.9% for control, *p* < 0.001 LOS, days, mean (SD): 1.0 (3.5) for intervention; 2.0 (3.5) for control, *p* = 0.840

*CI* confidence intervals, *CPS* clinical pharmacist specialist, *ED* emergency department, *IRR* incidence rate ratio, *LOS* length of stay, *OR* odds ratio, *PCP* primary care provider, *PT* physical therapist, *RCT* randomized control trial, *RN* registered nurse, *RR* risk ratio, *SD* standard deviation, *TAU* treatment as usual, *ZD* Zelen’s design

**Fig. 3:**
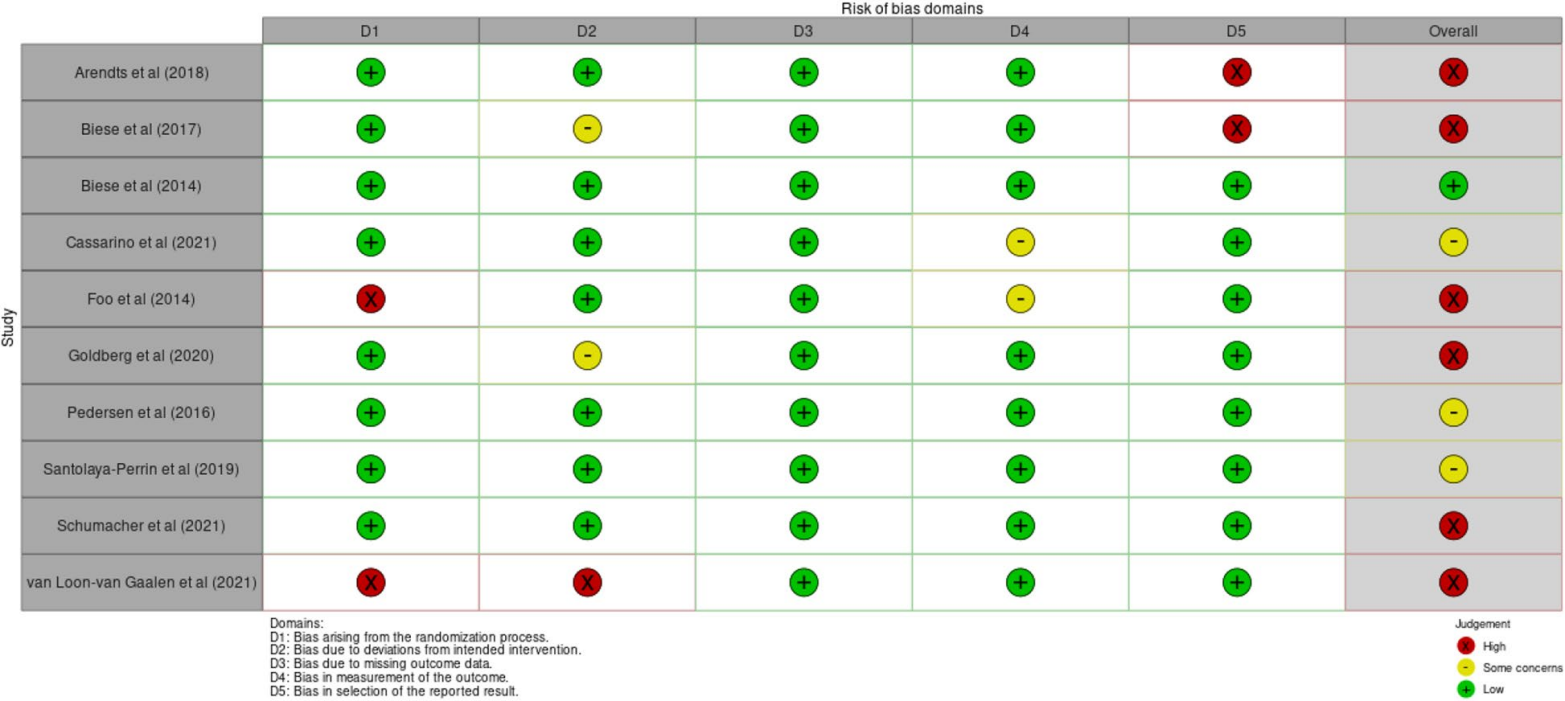
Risk of bias for controlled trials

**Fig. 4:**
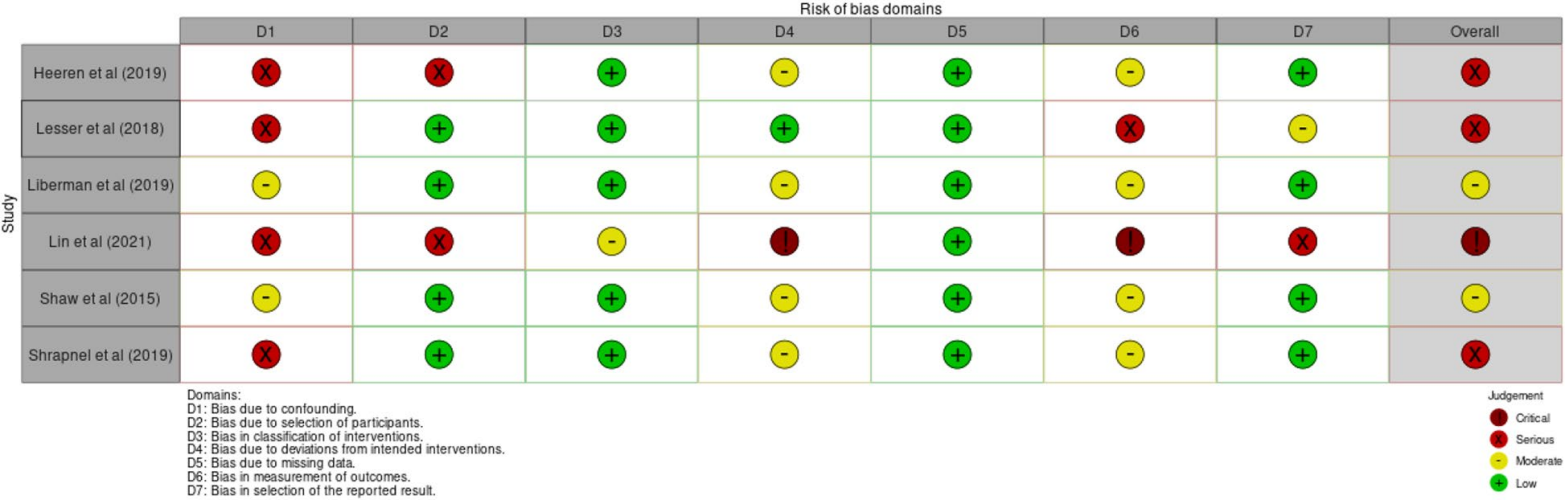
Risk of bias for observational studies

Study quality was moderate to low. Six controlled trials were assessed as having a high risk of bias [21, 23, 27, 31, 33, 35], three were assessed as having some concerns of bias [22, 30, 32], and one had a low risk of bias [20] (Fig. 3). One observational study was assessed as having a critical risk of bias [34], three had a serious risk of bias [24, 28, 29], and two had a moderate risk of bias [25, 26] (Fig. 4).

### Study population

Study population size ranged from 39 [20] to over 100,000 [24], with eight studies having a population over 1000 [21, 24, 26, 28–30, 33, 35] (Fig. 2, Table 2). Most studies (*n* = 10) included patients over 65 [20–27, 31, 32], two each included patients over 70 [29, 33] and over 75 [30, 34], and in two studies, the authors did not specify the age cut-off [28, 35]. Three studies included patients with other chronic conditions, such as chronic heart failure, chronic kidney disease, diabetes mellitus, or chronic obstructive pulmonary disease [32, 34, 35], and three required a specific acute condition for inclusion, such as a fall, urinary tract infection, or pneumonia [22, 23, 30]. Three studies specifically focused on patients who were considered “frequent users”, typically three or more ED visits in 12 months, or at high risk of reattendance [25, 27, 35]. Half of studies excluded patients who were living in a nursing home or other assisted living or were receiving palliative care [20, 21, 23, 27, 31, 33, 35].

### Interventions utilized

Several different interventions were utilized, half of which were multi-faceted. In total, 12 different interventions were assessed across the 16 studies: 5 interventions assessed follow-up telephone calls [20, 21, 27, 29, 33]; 4 assessed geriatric assessment, including comprehensive geriatric assessment [29–31, 34]; 4 assessed referrals [25, 27, 29, 31]; 3 assessed pharmacist-led interventions [23, 26, 32]; 2 assessed physical therapy services in the ED [23, 24]; 2 assessed care plans [25, 29]; 2 assessed education [25, 27]; 1 assessed case management [34]; 1 assessed home visits [30]; 1 assessed a care transition intervention [35]; 1 assessed a geriatric ED [26]; and 1 assessed care coordination [28]. Many of these interventions included similarities; for example care coordination and case management both typically involve someone from the ED reaching out to other care providers on behalf of the patient.

Four interventions assessed comprehensive geriatric assessment, a multidimensional process designed to assess the functional ability, health, social support, and environmental situation of older people to improve care [34]. Comprehensive geriatric assessment was implemented along with case management and care plans [34], home visits by a geriatrician post-discharge [30], care plans and referrals to a geriatric clinic [29], and referrals to community services and a geriatric clinic [31].

Four interventions involved a pharmacist or physical therapist in the ED. Two interventions were pharmacist-led, where a pharmacist reviewed patients’ prescriptions and made recommendations to the ED physicians on any necessary changes [26, 32]. One of these interventions was conducted in a geriatric-specific ED, which included environmental enhancements and geriatric training for staff [26]. One intervention assessed the impact of a physical therapist providing brief training and support to patients [24]. One intervention included both a pharmacist and a physical therapist present in the ED to provide support and advice to patients [23].

Five interventions assessed follow-up telephone calls, all of which included a nurse following up to ensure patients were following discharge instructions or to address any barriers patients were facing [20, 21, 27, 29, 33]. Three of these interventions assessed follow-up telephone calls as their only intervention [20, 21, 33]. Four interventions included referrals. The referrals consisted of a general referral of patients to community support or community-based geriatric support and did not include services to contact the supports or create appointments for patients [25, 27, 29, 31].. Two of these interventions also included care plans for patients [25, 29].

Two interventions assessed educational interventions in which patients were provided information on their health needs [25, 27]. One intervention assessed care coordination, where a clinical liaison ensured care was coordinated across the hospital and with patients’ primary care provider [28]. One assessed early assessment and intervention, where patients were assessed by a multidisciplinary team and a specific intervention was created based on patients’ needs [22]. Last, one intervention was a multi-faceted care transition intervention that included self-education, take-home plans for patients, and home visits when possible [35].

### Outcomes reported

**Fig. 5:**
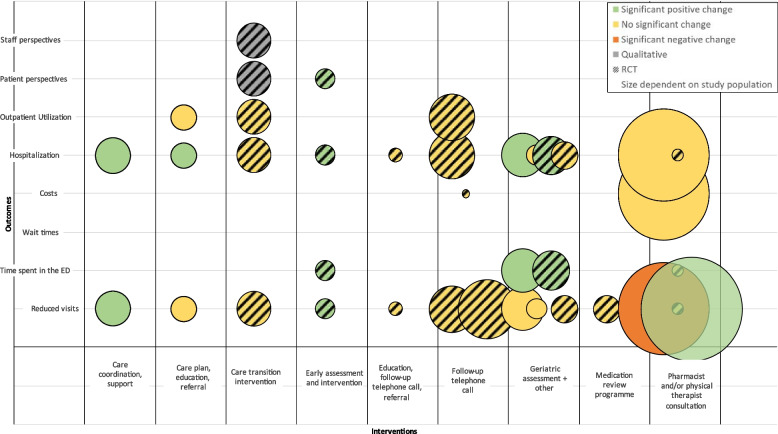
Effect of interventions for older adults. Legend: The interventions identified are across the *x*-axis, and the outcomes reported are on the *y*-axis. Each study is reported with one column of bubbles. The bubble size represents the size of the study

Three of 14 studies reported significant decreases in ED use, 1 assessing care coordination and support (*n* = 1121, serious risk of bias) [28], 1 assessing early assessment and intervention (*n* = 353, some concerns of bias) [22], and 1 assessing physical therapy services (*n* = 191,442, serious risk of bias) [24] (Fig. 5). One study assessing a geriatric-specific ED with support from a pharmacist reported a significant increase in ED revisits in intervention patients compared to control patients (*n* = 7864, moderate risk of bias) [26]. Five of 12 reported significant decreases in hospitalization [22, 25, 28–30] and 3 of 4 reported significant decreases in time spent in the ED [22, 29, 30] (Fig. 5). Neither study that reported on costs reported a significant change or difference in costs [20, 26], and none of the studies assessing outpatient utilization reported significant changes [21, 25, 35].

Care coordination with additional support and early assessment and intervention were the only two interventions that consistently reported improved outcomes for patients, though both studies had relatively small populations (*n* = 353 and *n* = 1121) [22, 28]. No study reporting on follow-up telephone calls reported any significant changes [20, 21, 27, 29, 33]. There were mixed outcomes for care plans, education, referrals, comprehensive geriatric assessment, and pharmacist or physical therapist consultations, with most reporting no significant changes.

### Sex and gender

Two studies reported on sex and/or gender differences, one assessing comprehensive geriatric assessment and case management [34] and one assessing follow-up phone calls [33]. In the geriatric assessment and case management intervention, older adults received individualized care plans based on comprehensive geriatric assessment [34]. Gender was included in the multivariate logistic regression model. Male gender was associated with decreased odds of admission following index ED visit [34]. In the other intervention, patients received a telephone call post-discharge to identify problems and offer additional guidance [33]. The authors examined the effects of the intervention on subgroups of patients at high risk for hospital return, including sex. There were no differences between males and females on unplanned ED revisit or hospitalization [33].

## Discussion

Sixteen studies of moderate-to-low quality were included. Overall, several different interventions were utilized for older adults, most of which did not report significant improvements in patient outcomes. Care coordination with additional support and early assessment and intervention were the only two interventions that consistently reported improved outcomes for patients, though both studies had relatively small populations. Of the two most common interventions, follow-up telephone calls and referrals, only two assessing referrals reported some significant changes, with both reporting reductions in hospitalization and one reporting reductions in time spent in the ED [25, 31]; no study reporting on follow-up telephone calls reported any significant changes. One intervention, a geriatric-specific ED with a clinical pharmacy specialist, reported significant increases in ED revisits in intervention patients compared to control patients [26]. No other study reported increased visits or significant negative outcomes.

There are still significant gaps in the literature on patient-related outcomes. Very few studies assessed time spent in the ED and outpatient utilization, and no study reported on wait times. Additionally, there was very limited information on the actual health outcomes of patients, and there is little information on whether these interventions improved non-ED-related outcomes. A similar review from 2019 reported small but significant improvements in some functional outcomes, despite also reporting few significant improvements in ED revisits or hospitalizations [9]. Additionally, a review assessing literature from 1985 to 2001 found that ED-based initiatives specifically for older adults report inconsistent success: ED revisit rates were not significantly different in the intervention groups compared to control groups, and some interventions reported increased hospitalizations in the intervention group [1]. Often, however, the reason for hospitalization was not discussed; increased hospitalization may be a positive outcome as it may mean that healthcare practitioners are thoroughly reviewing a patients’ needs and that patients’ needs are being addressed. Without additional information on the health status of patients, it is difficult to determine with the assessed outcomes whether patients’ needs are adequately being addressed.

Further, despite interventions not significantly improving ED-related outcomes, patients may have felt supported, which could lead to other benefits not assessed by this literature. Research has demonstrated that older adults tend to feel isolated, and decreasing these feelings of isolation has significant improvements on overall health and wellbeing [36]. Perhaps having additional contact with healthcare professionals reduces those feelings of isolation, leading to improved wellbeing. Patients may have felt like their concerns were being taken seriously, or that they were being cared for by involved professionals, ultimately leading to improvements in overall health and wellbeing. These outcomes, however, have not been assessed by this literature. Additionally, qualitative studies were excluded from this search, and only two studies reported on patient perspectives, so much is still unknown about patient views on the interventions.

As the focus of this review was on ED-based interventions, community-based, hospital-wide, or system-wide interventions not initiated in the ED were excluded but may have significant impacts on ED and overall healthcare use. Our review found that most ED-based interventions did not significantly reduce ED use by older adults, so wider-reaching interventions may be necessary to reduce the burden on the ED. However, it is important to understand the impact of ED-initiated and -based interventions to determine what EDs can implement themselves to support patients. ED administrators and physicians should understand which interventions are useful for older adults and can create interventions for their own EDs to attempt to help older patients.

It is clear from this literature that the healthcare needs of older adults are not being met in the ED or by ED-initiated interventions. As such, the focus of future work should be on other ways older adults’ needs can be met. The results of this review could suggest that most revisits in older adults are unavoidable, either due to frailty and disease trajectory, and efforts to support the unique care needs of older adults should focus elsewhere. Community-based primary care clinics, for example, may be better equipped to assist older adults; they may be better able to help older adults long term or provide more in-depth, comprehensive care than what the ED is able to provide. Additionally, many of the interventions identified in this review have been implemented repeatedly despite little evidence suggesting they are effective. Therefore, new, innovative interventions, multidisciplinary interventions, and collaboration with community and residential care facilities are needed to assist older adults and adequately address their needs.

## Conclusion

Most interventions identified by this review were not effective in reducing ED-related outcomes, and there are significant gaps in patient perspectives and the interventions’ effectiveness in addressing underlying health needs. Clearly, it is time for innovative interventions to support older adults both within and outside the ED.

### Supplementary Information

**Additional file 1. Appendix A.** Full text review search strategy

## Data Availability

All data generated or analysed during this study are included in this published article and its supplementary information files.

## Abbreviation

ED: Emergency department
