# Supplementary material for: Strategies for improving ED-related outcomes of older adults who seek care in emergency departments: a systematic review
Source: Int J Emerg Med. 2024 Feb 1;17:16. doi: 10.1186/s12245-024-00584-7 (PMC10835906; doi:10.1186/s12245-024-00584-7)
Supplement: Supplementary file 1 — Additional file 1. Appendix A. Full text review search strategy [file 12245_2024_584_MOESM1_ESM.docx]

**Appendix A: Full text review search strategy**

ED – Frequent Users

Final Strategies

2023 Jan 18

Ovid Multifile

Database: Embase <1974 to 2023 January 17>, Ovid MEDLINE(R) and Epub Ahead of Print, In-Process, In-Data-Review & Other Non-Indexed Citations and Daily <1946 to January 17, 2023>

Search Strategy:

--------------------------------------------------------------------------------

1 exp Emergency Service, Hospital/ (106013)

2 Emergency Medicine/ (60083)

3 emergency medicine.tw,kw,kf. (54372)

4 (emergency adj2 (center or centers or centre or centres or department? or dept or depts or room or rooms or unit or units or ward or wards)).tw,kw,kf. (383149)

5 (accident adj2 (center or centers or centre or centres or department? or dept or depts or room or rooms or unit or units or ward or wards)).tw,kw,kf. (1102)

6 ("A and E" adj2 (center or centers or centre or centres or department? or dept or depts or room or rooms or unit or units or ward or wards)).tw,kw,kf. (962)

7 (emergency adj (healthcare or health care or medical) adj service?).tw,kw,kf. (29883)

8 (hospital* adj3 emergency service?).tw,kw,kf. (4722)

9 (trauma adj (centre or centres or center or centers)).tw,kw,kf. (49074)

10 ((urgent or trauma) adj3 (care or healthcare or health care)).tw,kw,kf. (33357)

11 (ED adj5 emergenc*).tw,kw,kf. (87513)

12 or/1-11 [EMERGENCY DEPARTMENT, HOSPITAL EMERGENCY SERVICES] (556641)

13 *Patient Readmission/ (25743)

14 ((avoid* or avert* or circumvent* or decreas* or declin* or deter* or discourag* or dissuad* or lessen* or limit* or lower* or minimis* or minimiz* or prevent* or reduc*) adj3 ((repeat* adj3 attend*) or (repeat* adj3 visit*) or (return* adj3 attend*) or (return* adj3 visit*) or (frequen* adj3 attend*) or (frequen* adj3 visit*) or (unplanned adj3 attend*) or (unplanned adj3 return*) or (unplanned adj3 visit*) or (unscheduled adj3 attend*) or (unscheduled adj3 return*) or (unscheduled adj3 visit*) or (unanticipated adj3 attend*) or (unanticipated adj3 return*) or (unanticipated adj3 visit*) or (unforeseen adj3 attend*) or (unforeseen adj3 return*) or (unforeseen adj3 visit*) or revisit* or re-visit*)).tw,kw,kf. (3671)

15 (((avoid* or avert* or circumvent* or decreas* or declin* or deter* or discourag* or dissuad* or lessen* or limit* or lower* or minimis* or minimiz* or prevent* or reduc*) adj3 ((ED adj3 return*) or (EDs adj3 return*))) or (emergency department* adj3 return)).tw,kw,kf. (990)

16 (((before or inside or "less than" or "not greater than" or "prior to" or under or within) adj3 ("30" or thirty or one month or "1 month" or "the month" or four weeks or "4 weeks") adj3 ((ED adj3 return*) or (EDs adj3 return*))) or (emergency department* adj3 return)).tw,kw,kf. (1062)

17 (((before or inside or "less than" or "not greater than" or "prior to" or under or within) adj3 ("30" or thirty or one month or "1 month" or "the month" or four weeks or "4 weeks") adj3 ((ED adj3 revisit*) or (EDs adj3 revisit*))) or (emergency department* adj3 revisit*)).tw,kw,kf. (706)

18 (((before or inside or "less than" or "not greater than" or "prior to" or under or within) adj3 ("30" or thirty or one month or "1 month" or "the month" or four weeks or "4 weeks") adj3 ((ED adj3 re-visit*) or (EDs adj3 re-visit*))) or (emergency department* adj3 re-visit*)).tw,kw,kf. (24)

19 ((avoid* or avert* or circumvent* or decreas* or declin* or deter* or discourag* or dissuad* or lessen* or limit* or lower* or minimis* or minimiz* or prevent* or reduc*) adj3 ((ED adj3 attend*) or (EDs adj3 attend*) or (ED adj3 visit*) or (EDs adj3 visit*) or (emergency department* adj3 attend*) or (emergency department* adj3 visit*))).tw,kw,kf. (6695)

20 ((avoid* or avert* or circumvent* or decreas* or declin* or deter* or discourag* or dissuad* or lessen* or limit* or lower* or minimis* or minimiz* or prevent* or reduc*) adj5 ("use" or used or user or users or uses or using or utilis* or utiliz*) adj3 (visit* or attend*)).tw,kw,kf. (2574)

21 ((avoid* or avert* or circumvent* or decreas* or declin* or deter* or discourag* or dissuad* or lessen* or limit* or lower* or minimis* or minimiz* or prevent* or reduc*) adj3 (misuse or misused or misuses or misusing or misuser? or mis-use or mis-used or mis-uses or mis-using or mis-user?)).tw,kw,kf. (4528)

22 ((avoid* or avert* or circumvent* or decreas* or declin* or deter* or discourag* or dissuad* or lessen* or limit* or lower* or minimis* or minimiz* or prevent* or reduc*) adj3 (overuse or overused or overuses or overusing or overuser? or over-use or over-used or over-uses or over-using or over-user? or overutilis* or over-utilis* or overutiliz* or over-utiliz*)).tw,kw,kf. (4620)

23 ((avoid* or avert* or circumvent* or decreas* or declin* or deter* or discourag* or dissuad* or lessen* or limit* or lower* or minimis* or minimiz* or prevent* or reduc*) adj3 (superuse or superused or superuses or superusing or superuser? or super-use or super-used or super-uses or super-using or super-user? or superutilis* or super-utilis* or superutiliz* or super-utiliz*)).tw,kw,kf. (30)

24 ((avoid* or avert* or circumvent* or decreas* or declin* or deter* or discourag* or dissuad* or lessen* or limit* or lower* or minimis* or minimiz* or prevent* or reduc*) adj3 (hyperuse* or hyperusing or hyper-use* or hyper-using)).tw,kw,kf. (0)

25 (hotspot* or hot-spot*).tw,kw,kf. (94062)

26 (((((return* adj2 visit*) or (return* adj2 attend*) or (repeat* adj2 visit*) or (repeat* adj2 attend*) or revisit* or re-visit*) and (post or after or following* or home or initial* or newly or recent*)) adj3 discharg*) or postdischarg* or post-ED or post-hospitali#ation?).tw,kw,kf. (16553)

27 or/13-26 [REDUCE ATTENDANCE/VISITS, HOTSPOTTING] (156436)

28 12 and 27 [ED - REDUCE ATTENDANCE/VISITS, HOTSPOTTING] (14884)

29 exp Animals/ not Humans/ (16609523)

30 28 not 29 [ANIMAL-ONLY REMOVED] (12400)

31 "Utilization Review"/ (67794)

32 "Concurrent Review"/ (60032)

33 ((utili#ation or concurrent) adj review?).tw,kw,kf. (4182)

34 Evaluation Study.pt. (261753)

35 (evaluation adj (study or studies)).tw,kw,kf. (18098)

36 (intervention* adj (study or studies)).tw,kw,kf. (85248)

37 (intervention* or initiative? or innovat*).ti,kw,kf. (677909)

38 (intervention* or initiative? or innovat*).ab. /freq=2 (1044105)

39 or/31-38 (1784688)

40 30 and 39 [ED - FREQUENT USERS - UTILIZATION/CONCURRENT REVIEWS, EVALUATION STUDIES] (2417)

41 (controlled clinical trial or randomized controlled trial or pragmatic clinical trial or equivalence trial).pt. (676478)

42 "Clinical Trials as Topic"/ (293340)

43 exp "Controlled Clinical Trials as Topic"/ (421783)

44 (randomi#ed or randomi#ation? or randomly or RCT or placebo*).tw,kw,kf. (2847236)

45 ((singl* or doubl* or trebl* or tripl*) adj (mask* or blind* or dumm*)).tw,kw,kf. (467534)

46 trial.ti. (657923)

47 or/41-46 (3611750)

48 30 and 47 [ED - FREQUENT USERS - RCTs] (1342)

49 controlled clinical trial.pt. (95158)

50 Controlled Clinical Trial/ or Controlled Clinical Trials as Topic/ (579944)

51 (control* adj2 trial).tw,kw,kf. (476947)

52 Non-Randomized Controlled Trials as Topic/ (13841)

53 (nonrandom* or non-random* or quasi-random* or quasi-experiment*).tw,kw,kf. (160495)

54 (nRCT or non-RCT).tw,kw,kf. (1142)

55 Controlled Before-After Studies/ (229052)

56 (control* adj3 ("before and after" or "before after")).tw,kw,kf. (11827)

57 Interrupted Time Series Analysis/ (222368)

58 time series.tw,kw,kf. (92422)

59 (pre- adj5 post-).tw,kw,kf. (347798)

60 ((pretest adj5 posttest) or (pre-test adj5 post-test)).tw,kw,kf. (26303)

61 Historically Controlled Study/ (239458)

62 (control* adj2 study).tw,kw,kf. (484074)

63 Control Groups/ (112478)

64 (control* adj2 group?).tw,kf. (1471740)

65 trial.ti. (657923)

66 or/49-65 (3718181)

67 30 and 66 [ED - FREQUENT USERS - nRCTs] (2121)

68 exp Cohort Studies/ (3383032)

69 cohort?.tw,kw,kf. (2192279)

70 Retrospective Studies/ (2176773)

71 (longitudinal or prospective or retrospective).tw,kw,kf. (4118774)

72 ((followup or follow-up) adj (study or studies)).tw,kw,kf. (134701)

73 Observational study.pt. (136961)

74 (observation$2 adj (study or studies)).tw,kw,kf. (397526)

75 ((population or population-based) adj (study or studies or analys#s)).tw,kw,kf. (56679)

76 ((multidimensional or multi-dimensional) adj (study or studies)).tw,kw,kf. (325)

77 Comparative Study.pt. (1911937)

78 ((comparative or comparison) adj (study or studies)).tw,kw,kf. (285074)

79 exp Case-Control Studies/ (1599446)

80 ((case-control* or case-based or case-comparison or case-compeer or case-referrent or case-referent) adj3 (study or studies)).tw,kw,kf. (317236)

81 Cross-Sectional Studies/ (855062)

82 (crosssection* or cross-section*).tw,kw,kf. (1222620)

83 or/68-82 (10419821)

84 30 and 83 [ED - FREQUENT USERS - OBSERVATIONAL STUDIES] (7262)

85 40 or 48 or 67 or 84 [ALL STUDY DESIGNS] (9219)

86 (exp Child/ or exp Infant/ or Adolescent/) not exp Adult/ (4523754)

87 85 not 86 [CHILD-, INFANT-ONLY REMOVED] (7798)

88 limit 87 to yr="2013-current" (6545)

89 88 use ppez [MEDLINE RECORDS] (3058)

90 hospital emergency service/ (94229)

91 emergency ward/ (281451)

92 emergency medicine/ (60083)

93 emergency medicine.tw,kw,kf. (54372)

94 (emergency adj2 (center or centers or centre or centres or department? or dept or depts or room or rooms or unit or units or ward or wards)).tw,kw,kf. (383149)

95 (accident adj2 (center or centers or centre or centres or department? or dept or depts or room or rooms or unit or units or ward or wards)).tw,kw,kf. (1102)

96 ("A and E" adj2 (center or centers or centre or centres or department? or dept or depts or room or rooms or unit or units or ward or wards)).tw,kw,kf. (962)

97 (emergency adj (healthcare or health care or medical) adj service?).tw,kw,kf. (29883)

98 (hospital* adj3 emergency service?).tw,kw,kf. (4722)

99 (trauma adj (centre or centres or center or centers)).tw,kw,kf. (49074)

100 ((urgent or trauma) adj3 (care or healthcare or health care)).tw,kw,kf. (33357)

101 (ED adj5 emergenc*).tw,kw,kf. (87513)

102 or/90-101 [EMERGENCY DEPARTMENT, HOSPITAL EMERGENCY SERVICES] (595726)

103 *hospital readmission/ (28129)

104 ((avoid* or avert* or circumvent* or decreas* or declin* or deter* or discourag* or dissuad* or lessen* or limit* or lower* or minimis* or minimiz* or prevent* or reduc*) adj3 ((repeat* adj3 attend*) or (repeat* adj3 visit*) or (return* adj3 attend*) or (return* adj3 visit*) or (frequen* adj3 attend*) or (frequen* adj3 visit*) or (unplanned adj3 attend*) or (unplanned adj3 return*) or (unplanned adj3 visit*) or (unscheduled adj3 attend*) or (unscheduled adj3 return*) or (unscheduled adj3 visit*) or (unanticipated adj3 attend*) or (unanticipated adj3 return*) or (unanticipated adj3 visit*) or (unforeseen adj3 attend*) or (unforeseen adj3 return*) or (unforeseen adj3 visit*) or revisit* or re-visit*)).tw,kw,kf. (3671)

105 (((avoid* or avert* or circumvent* or decreas* or declin* or deter* or discourag* or dissuad* or lessen* or limit* or lower* or minimis* or minimiz* or prevent* or reduc*) adj3 ((ED adj3 return*) or (EDs adj3 return*))) or (emergency department* adj3 return)).tw,kw,kf. (990)

106 (((before or inside or "less than" or "not greater than" or "prior to" or under or within) adj3 ("30" or thirty or one month or "1 month" or "the month" or four weeks or "4 weeks") adj3 ((ED adj3 return*) or (EDs adj3 return*))) or (emergency department* adj3 return)).tw,kw,kf. (1062)

107 (((before or inside or "less than" or "not greater than" or "prior to" or under or within) adj3 ("30" or thirty or one month or "1 month" or "the month" or four weeks or "4 weeks") adj3 ((ED adj3 revisit*) or (EDs adj3 revisit*))) or (emergency department* adj3 revisit*)).tw,kw,kf. (706)

108 (((before or inside or "less than" or "not greater than" or "prior to" or under or within) adj3 ("30" or thirty or one month or "1 month" or "the month" or four weeks or "4 weeks") adj3 ((ED adj3 re-visit*) or (EDs adj3 re-visit*))) or (emergency department* adj3 re-visit*)).tw,kw,kf. (24)

109 ((avoid* or avert* or circumvent* or decreas* or declin* or deter* or discourag* or dissuad* or lessen* or limit* or lower* or minimis* or minimiz* or prevent* or reduc*) adj3 ((ED adj3 attend*) or (EDs adj3 attend*) or (ED adj3 visit*) or (EDs adj3 visit*) or (emergency department* adj3 attend*) or (emergency department* adj3 visit*))).tw,kw,kf. (6695)

110 ((avoid* or avert* or circumvent* or decreas* or declin* or deter* or discourag* or dissuad* or lessen* or limit* or lower* or minimis* or minimiz* or prevent* or reduc*) adj5 ("use" or used or user or users or uses or using or utilis* or utiliz*) adj3 (visit* or attend*)).tw,kw,kf. (2574)

111 ((avoid* or avert* or circumvent* or decreas* or declin* or deter* or discourag* or dissuad* or lessen* or limit* or lower* or minimis* or minimiz* or prevent* or reduc*) adj3 (misuse or misused or misuses or misusing or misuser? or mis-use or mis-used or mis-uses or mis-using or mis-user?)).tw,kw,kf. (4528)

112 ((avoid* or avert* or circumvent* or decreas* or declin* or deter* or discourag* or dissuad* or lessen* or limit* or lower* or minimis* or minimiz* or prevent* or reduc*) adj3 (overuse or overused or overuses or overusing or overuser? or over-use or over-used or over-uses or over-using or over-user? or overutilis* or over-utilis* or overutiliz* or over-utiliz*)).tw,kw,kf. (4620)

113 ((avoid* or avert* or circumvent* or decreas* or declin* or deter* or discourag* or dissuad* or lessen* or limit* or lower* or minimis* or minimiz* or prevent* or reduc*) adj3 (superuse or superused or superuses or superusing or superuser? or super-use or super-used or super-uses or super-using or super-user? or superutilis* or super-utilis* or superutiliz* or super-utiliz*)).tw,kw,kf. (30)

114 ((avoid* or avert* or circumvent* or decreas* or declin* or deter* or discourag* or dissuad* or lessen* or limit* or lower* or minimis* or minimiz* or prevent* or reduc*) adj3 (hyperuse* or hyperusing or hyper-use* or hyper-using)).tw,kw,kf. (0)

115 (hotspot* or hot-spot*).tw,kw,kf. (94062)

116 (((((return* adj2 visit*) or (return* adj2 attend*) or (repeat* adj2 visit*) or (repeat* adj2 attend*) or revisit* or re-visit*) and (post or after or following* or home or initial* or newly or recent*)) adj3 discharg*) or postdischarg* or post-ED or post-hospitali#ation?).tw,kw,kf. (16553)

117 or/103-116 [REDUCE ATTENDANCE/VISITS, HOTSPOTTING] (158675)

118 102 and 117 [ED - REDUCE ATTENDANCE/VISITS, HOTSPOTTING] (15254)

119 (exp animal/ or exp animal experimentation/ or exp animal model/ or exp animal experiment/ or nonhuman/ or exp vertebrate/) not (exp human/ or exp human experimentation/ or exp human experiment/) (12167374)

120 118 not 119 [ANIMAL-ONLY REMOVED] (15237)

121 utilization review/ (67794)

122 ((utili#ation or concurrent) adj review?).tw,kw,kf. (4182)

123 exp evaluation study/ (351246)

124 (evaluation adj (study or studies)).tw,kw,kf. (18098)

125 (intervention* adj (study or studies)).tw,kw,kf. (85248)

126 (intervention* or initiative? or innovat*).ti,kw,kf. (677909)

127 (intervention* or initiative? or innovat*).ab. /freq=2 (1044105)

128 or/121-127 (1859185)

129 120 and 128 [ED - FREQUENT USERS - UTILIZATION/CONCURRENT REVIEWS, EVALUATION STUDIES] (3087)

130 exp randomized controlled trial/ or controlled clinical trial/ (1615821)

131 clinical trial/ (1589739)

132 exp "controlled clinical trial (topic)"/ (252332)

133 (randomi#ed or randomi#ation? or randomly or RCT or placebo*).tw,kw,kf. (2847236)

134 ((singl* or doubl* or trebl* or tripl*) adj (mask* or blind* or dumm*)).tw,kw,kf. (467534)

135 trial.ti. (657923)

136 or/130-135 (4312154)

137 120 and 136 [ED - FREQUENT USERS - RCTs] (1918)

138 controlled clinical trial/ (563222)

139 "controlled clinical trial (topic)"/ (12787)

140 (control* adj2 trial).tw,kw,kf. (476947)

141 (nonrandom* or non-random* or quasi-random* or quasi-experiment*).tw,kw,kf. (160495)

142 (nRCT or non-RCT).tw,kw,kf. (1142)

143 (control* adj3 ("before and after" or "before after")).tw,kw,kf. (11827)

144 time series analysis/ (35253)

145 time series.tw,kw,kf. (92422)

146 pretest posttest control group design/ (634)

147 (pre- adj5 post-).tw,kw,kf. (347798)

148 ((pretest adj5 posttest) or (pre-test adj5 post-test)).tw,kw,kf. (26303)

149 controlled study/ (9342411)

150 (control* adj2 study).tw,kw,kf. (484074)

151 control group/ (112478)

152 (control* adj2 group?).tw,kf. (1471740)

153 trial.ti. (657923)

154 or/138-153 (11462683)

155 120 and 154 [ED - FREQUENT USERS - nRCTs] (5280)

156 cohort analysis/ (1270306)

157 cohort?.tw,kw,kf. (2192279)

158 retrospective study/ (2456170)

159 longitudinal study/ (347022)

160 prospective study/ (1471828)

161 (longitudinal or prospective or retrospective).tw,kw,kf. (4118774)

162 follow up/ (1952863)

163 ((followup or follow-up) adj (study or studies)).tw,kw,kf. (134701)

164 observational study/ (441102)

165 (observation$2 adj (study or studies)).tw,kw,kf. (397526)

166 population research/ (128699)

167 ((population or population-based) adj (study or studies or analys#s)).tw,kw,kf. (56679)

168 ((multidimensional or multi-dimensional) adj (study or studies)).tw,kw,kf. (325)

169 exp comparative study/ (3527803)

170 ((comparative or comparison) adj (study or studies)).tw,kw,kf. (285074)

171 exp case control study/ (1599446)

172 ((case-control* or case-based or case-comparison or case-compeer or case-referrent or case-referent) adj3 (study or studies)).tw,kw,kf. (317236)

173 cross-sectional study/ (983334)

174 (crosssection* or cross-section*).tw,kw,kf. (1222620)

175 major clinical study/ (4736403)

176 or/156-175 (14890787)

177 120 and 176 [ED - FREQUENT USERS - OBSERVATIONAL STUDIES] (10832)

178 129 or 137 or 155 or 177 [ALL STUDY DESIGNS] (12664)

179 (juvenile/ or exp adolescent/ or exp child/) not exp adult/ (4039524)

180 178 not 179 [UNDER 18 POPULATION-ONLY REMOVED] (10882)

181 conference abstract.pt. (4661419)

182 180 not 181 (8145)

183 180 and 181 (2737)

184 limit 183 to yr="2021-current" (515)

185 182 or 184 [MOST RECENT 2 YEARS CONFERENCE ABSTRACTS RETAINED] (8660)

186 limit 185 to yr="2013-current" [DATE LIMIT APPLIED] (7094)

187 186 use oemezd [EMBASE RECORDS] (4023)

188 89 or 187 [BOTH DATABASES] (7081)

189 limit 188 to yr="2019-current" (4192)

190 remove duplicates from 189 (2742)

191 188 not 189 (2889)

192 remove duplicates from 191 (1785)

193 190 or 192 [T0TAL UNIQUE RECORDS] (4527)

194 193 use ppez [MEDLINE UNIQUE RECORDS] (3049)

195 193 use oemezd [EMBASE UNIQUE RECORDS] (1478)

***************************

CINAHL

| # | Query | Limiters/Expanders | Last Run Via | Results |
| --- | --- | --- | --- | --- |
| S82 | S36 OR S44 OR S62 OR S79 | Limiters - Published Date: 20130101-20231231  Search modes - Find all my search terms | Interface - EBSCOhost Research Databases  Search Screen - Advanced Search  Database - CINAHL Plus with Full Text | 2,302 |
| S81 | S36 OR S44 OR S62 OR S79 | Search modes - Find all my search terms | Interface - EBSCOhost Research Databases  Search Screen - Advanced Search  Database - CINAHL Plus with Full Text | 2,913 |
| S80 | S36 OR S44 OR S62 OR S79 | Search modes - Find all my search terms | Interface - EBSCOhost Research Databases  Search Screen - Advanced Search  Database - CINAHL Plus with Full Text | 2,913 |
| S79 | S28 AND S78 | Search modes - Find all my search terms | Interface - EBSCOhost Research Databases  Search Screen - Advanced Search  Database - CINAHL Plus with Full Text | 2,400 |
| S78 | S63 OR S64 OR S65 OR S66 OR S67 OR S68 OR S69 OR S70 OR S71 OR S72 OR S73 OR S74 OR S75 OR S76 OR S77 | Search modes - Find all my search terms | Interface - EBSCOhost Research Databases  Search Screen - Advanced Search  Database - CINAHL Plus with Full Text | 1,528,233 |
| S77 | TI ( crosssection* or (cross W0 section*) ) OR AB ( crosssection* or (cross W0 section*) ) | Search modes - Find all my search terms | Interface - EBSCOhost Research Databases  Search Screen - Advanced Search  Database - CINAHL Plus with Full Text | 206,987 |
| S76 | (MH "Cross Sectional Studies") | Search modes - Find all my search terms | Interface - EBSCOhost Research Databases  Search Screen - Advanced Search  Database - CINAHL Plus with Full Text | 243,837 |
| S75 | TI ( ((case W0 control*) or "case-based" or "case-comparison" or "case-compeer" or "case-referrent" or "case-referent") N3 (study or studies) ) OR AB ( ((case W0 control*) or "case-based" or "case-comparison" or "case-compeer" or "case-referrent" or "case-referent") N3 (study or studies) ) | Search modes - Find all my search terms | Interface - EBSCOhost Research Databases  Search Screen - Advanced Search  Database - CINAHL Plus with Full Text | 38,265 |
| S74 | (MH "Case Control Studies+") | Search modes - Find all my search terms | Interface - EBSCOhost Research Databases  Search Screen - Advanced Search  Database - CINAHL Plus with Full Text | 92,854 |
| S73 | TI ( (comparative or comparison) W0 (study or studies) ) OR AB ( (comparative or comparison) W0 (study or studies) ) | Search modes - Find all my search terms | Interface - EBSCOhost Research Databases  Search Screen - Advanced Search  Database - CINAHL Plus with Full Text | 20,487 |
| S72 | (MH "Comparative Studies") | Search modes - Find all my search terms | Interface - EBSCOhost Research Databases  Search Screen - Advanced Search  Database - CINAHL Plus with Full Text | 457,781 |
| S71 | TI ( (multidimensional or "multi-dimensional") W0 (study or studies or analys?s) ) OR AB ( (multidimensional or "multi-dimensional") W0 (study or studies or analys?s) ) | Search modes - Find all my search terms | Interface - EBSCOhost Research Databases  Search Screen - Advanced Search  Database - CINAHL Plus with Full Text | 193 |
| S70 | TI ( (population or "population-based") W0 (study or studies or analys?s) ) OR AB ( (population or "population-based") W0 (study or studies or analys?s) ) | Search modes - Find all my search terms | Interface - EBSCOhost Research Databases  Search Screen - Advanced Search  Database - CINAHL Plus with Full Text | 22,839 |
| S69 | TI ( observation* W0 (study or studies) ) OR AB ( observation* W0 (study or studies) ) | Search modes - Find all my search terms | Interface - EBSCOhost Research Databases  Search Screen - Advanced Search  Database - CINAHL Plus with Full Text | 57,313 |
| S68 | (MH "Nonexperimental Studies") | Search modes - Find all my search terms | Interface - EBSCOhost Research Databases  Search Screen - Advanced Search  Database - CINAHL Plus with Full Text | 33,451 |
| S67 | TI ( (followup or follow-up) adj (study or studies) ) OR AB ( (followup or follow-up) adj (study or studies) ) | Search modes - Find all my search terms | Interface - EBSCOhost Research Databases  Search Screen - Advanced Search  Database - CINAHL Plus with Full Text | 79 |
| S66 | TI ( longitudinal or prospective or retrospective ) OR AB ( longitudinal or prospective or retrospective ) | Search modes - Find all my search terms | Interface - EBSCOhost Research Databases  Search Screen - Advanced Search  Database - CINAHL Plus with Full Text | 509,815 |
| S65 | (MH "Prospective Studies+") | Search modes - Find all my search terms | Interface - EBSCOhost Research Databases  Search Screen - Advanced Search  Database - CINAHL Plus with Full Text | 518,555 |
| S64 | (MH "Retrospective Panel Studies") | Search modes - Find all my search terms | Interface - EBSCOhost Research Databases  Search Screen - Advanced Search  Database - CINAHL Plus with Full Text | 200 |
| S63 | TI cohort# OR AB cohort# | Search modes - Find all my search terms | Interface - EBSCOhost Research Databases  Search Screen - Advanced Search  Database - CINAHL Plus with Full Text | 286,971 |
| S62 | S28 AND S61 | Search modes - Find all my search terms | Interface - EBSCOhost Research Databases  Search Screen - Advanced Search  Database - CINAHL Plus with Full Text | 804 |
| S61 | S45 OR S46 OR S47 OR S48 OR S49 OR S50 OR S51 OR S52 OR S53 OR S54 OR S55 OR S56 OR S57 OR S58 OR S59 OR S60 | Search modes - Find all my search terms | Interface - EBSCOhost Research Databases  Search Screen - Advanced Search  Database - CINAHL Plus with Full Text | 523,480 |
| S60 | TI trial | Search modes - Find all my search terms | Interface - EBSCOhost Research Databases  Search Screen - Advanced Search  Database - CINAHL Plus with Full Text | 174,857 |
| S59 | TI control* N2 group# OR AB control* N2 group# | Search modes - Find all my search terms | Interface - EBSCOhost Research Databases  Search Screen - Advanced Search  Database - CINAHL Plus with Full Text | 147,370 |
| S58 | (MH "Control Group") | Search modes - Find all my search terms | Interface - EBSCOhost Research Databases  Search Screen - Advanced Search  Database - CINAHL Plus with Full Text | 13,488 |
| S57 | TI control* N2 study OR AB control* N2 study | Search modes - Find all my search terms | Interface - EBSCOhost Research Databases  Search Screen - Advanced Search  Database - CINAHL Plus with Full Text | 91,680 |
| S56 | (MH "Historically Controlled Study") | Search modes - Find all my search terms | Interface - EBSCOhost Research Databases  Search Screen - Advanced Search  Database - CINAHL Plus with Full Text | 143 |
| S55 | (MH "Pretest-Posttest Design+") | Search modes - Find all my search terms | Interface - EBSCOhost Research Databases  Search Screen - Advanced Search  Database - CINAHL Plus with Full Text | 52,911 |
| S54 | TI ( (pretest N5 posttest) or ("pre-test" N5 "post-test") ) OR AB ( (pretest N5 posttest) or ("pre-test" N5 "post-test") ) | Search modes - Find all my search terms | Interface - EBSCOhost Research Databases  Search Screen - Advanced Search  Database - CINAHL Plus with Full Text | 8,963 |
| S53 | TI "pre-" N5 "post-" OR AB "pre-" N5 "post-" | Search modes - Find all my search terms | Interface - EBSCOhost Research Databases  Search Screen - Advanced Search  Database - CINAHL Plus with Full Text | 46,000 |
| S52 | TI "time series" OR AB "time series" | Search modes - Find all my search terms | Interface - EBSCOhost Research Databases  Search Screen - Advanced Search  Database - CINAHL Plus with Full Text | 7,859 |
| S51 | (MH "Interrupted Time Series Analysis") | Search modes - Find all my search terms | Interface - EBSCOhost Research Databases  Search Screen - Advanced Search  Database - CINAHL Plus with Full Text | 858 |
| S50 | TI ( control* N3 ("before and after" or "before after") ) OR AB ( control* N3 ("before and after" or "before after") ) | Search modes - Find all my search terms | Interface - EBSCOhost Research Databases  Search Screen - Advanced Search  Database - CINAHL Plus with Full Text | 2,092 |
| S49 | (MH "Controlled Before-After Studies") | Search modes - Find all my search terms | Interface - EBSCOhost Research Databases  Search Screen - Advanced Search  Database - CINAHL Plus with Full Text | 232 |
| S48 | (MH "Nonrandomized Trials") | Search modes - Find all my search terms | Interface - EBSCOhost Research Databases  Search Screen - Advanced Search  Database - CINAHL Plus with Full Text | 848 |
| S47 | TI ( nRCT or "non-RCT" ) OR AB ( nRCT or "non-RCT" ) | Search modes - Find all my search terms | Interface - EBSCOhost Research Databases  Search Screen - Advanced Search  Database - CINAHL Plus with Full Text | 211 |
| S46 | TI ( nonrandom* or (non W0 random*) or (quasi W0 random*) or (quasi W0 experiment*) ) OR AB ( nonrandom* or (non W0 random*) or (quasi W0 random*) or (quasi W0 experiment*) ) | Search modes - Find all my search terms | Interface - EBSCOhost Research Databases  Search Screen - Advanced Search  Database - CINAHL Plus with Full Text | 28,361 |
| S45 | TI control* N2 trial OR AB control* N2 trial | Search modes - Find all my search terms | Interface - EBSCOhost Research Databases  Search Screen - Advanced Search  Database - CINAHL Plus with Full Text | 165,142 |
| S44 | S28 AND S43 | Search modes - Find all my search terms | Interface - EBSCOhost Research Databases  Search Screen - Advanced Search  Database - CINAHL Plus with Full Text | 514 |
| S43 | S37 OR S38 OR S39 OR S40 OR S41 OR S42 | Search modes - Find all my search terms | Interface - EBSCOhost Research Databases  Search Screen - Advanced Search  Database - CINAHL Plus with Full Text | 486,088 |
| S42 | TI trial | Search modes - Find all my search terms | Interface - EBSCOhost Research Databases  Search Screen - Advanced Search  Database - CINAHL Plus with Full Text | 174,857 |
| S41 | TI ( ((singl* or doubl* or trebl* or tripl*) W0 (mask* or blind* or dumm*) ) OR AB ( ((singl* or doubl* or trebl* or tripl*) W0 (mask* or blind* or dumm*) ) | Search modes - Find all my search terms | Interface - EBSCOhost Research Databases  Search Screen - Advanced Search  Database - CINAHL Plus with Full Text | 57,940 |
| S40 | TI ( randomi?ed or randomi?ation# or randomly or RCT or placebo* ) OR AB ( randomi?ed or randomi?ation# or randomly or RCT or placebo* ) | Search modes - Find all my search terms | Interface - EBSCOhost Research Databases  Search Screen - Advanced Search  Database - CINAHL Plus with Full Text | 398,604 |
| S39 | (MH "Double-Blind Studies") OR (MH "Single-Blind Studies") OR (MH "Triple-Blind Studies") | Search modes - Find all my search terms | Interface - EBSCOhost Research Databases  Search Screen - Advanced Search  Database - CINAHL Plus with Full Text | 69,893 |
| S38 | (MH "Intervention Trials") | Search modes - Find all my search terms | Interface - EBSCOhost Research Databases  Search Screen - Advanced Search  Database - CINAHL Plus with Full Text | 7,769 |
| S37 | (MH "Randomized Controlled Trials+") | Search modes - Find all my search terms | Interface - EBSCOhost Research Databases  Search Screen - Advanced Search  Database - CINAHL Plus with Full Text | 136,384 |
| S36 | S28 AND S35 | Search modes - Find all my search terms | Interface - EBSCOhost Research Databases  Search Screen - Advanced Search  Database - CINAHL Plus with Full Text | 723 |
| S35 | S29 OR S30 OR S31 OR S32 OR S33 OR S34 | Search modes - Find all my search terms | Interface - EBSCOhost Research Databases  Search Screen - Advanced Search  Database - CINAHL Plus with Full Text | 509,304 |
| S34 | TI intervention* or initiative# or innovat* | Search modes - Find all my search terms | Interface - EBSCOhost Research Databases  Search Screen - Advanced Search  Database - CINAHL Plus with Full Text | 156,302 |
| S33 | TI ( intervention W0 (study or studies) ) OR AB ( intervention W0 (study or studies) ) | Search modes - Find all my search terms | Interface - EBSCOhost Research Databases  Search Screen - Advanced Search  Database - CINAHL Plus with Full Text | 11,508 |
| S32 | TI ( evaluation W0 (study or studies) ) OR AB ( evaluation W0 (study or studies) ) | Search modes - Find all my search terms | Interface - EBSCOhost Research Databases  Search Screen - Advanced Search  Database - CINAHL Plus with Full Text | 2,938 |
| S31 | (MH "Evaluation Research+") | Search modes - Find all my search terms | Interface - EBSCOhost Research Databases  Search Screen - Advanced Search  Database - CINAHL Plus with Full Text | 351,662 |
| S30 | TI ( (utili?ation or concurrent) W0 review# ) OR AB ( (utili?ation or concurrent) W0 review# ) | Search modes - Find all my search terms | Interface - EBSCOhost Research Databases  Search Screen - Advanced Search  Database - CINAHL Plus with Full Text | 621 |
| S29 | (MH "Utilization Review") | Search modes - Find all my search terms | Interface - EBSCOhost Research Databases  Search Screen - Advanced Search  Database - CINAHL Plus with Full Text | 2,198 |
| S28 | S12 AND S27 | Search modes - Find all my search terms | Interface - EBSCOhost Research Databases  Search Screen - Advanced Search  Database - CINAHL Plus with Full Text | 4,426 |
| S27 | S13 OR S14 OR S15 OR S16 OR S17 OR S18 OR S19 OR S20 OR S21 OR S22 OR S23 OR S24 OR S25 OR S26 | Search modes - Find all my search terms | Interface - EBSCOhost Research Databases  Search Screen - Advanced Search  Database - CINAHL Plus with Full Text | 21,007 |
| S26 | TI ( (((((return* N2 visit*) or (return* N2 attend*) or (repeat* N2 visit*) or (repeat* N2 attend*) or revisit* or re-visit*) and (post or after or following* or home or initial* or newly or recent*)) N3 discharg*) or postdischarg* or "post-ED" or post-hospitali?ation#) ) OR AB ( (((((return* N2 visit*) or (return* N2 attend*) or (repeat* N2 visit*) or (repeat* N2 attend*) or revisit* or re-visit*) and (post or after or following* or home or initial* or newly or recent*)) N3 discharg*) or postdischarg* or "post-ED" or post-hospitali?ation#) ) | Search modes - Find all my search terms | Interface - EBSCOhost Research Databases  Search Screen - Advanced Search  Database - CINAHL Plus with Full Text | 3,945 |
| S25 | TI ( hotspot* or (hot W0 spot*) ) OR AB ( hotspot* or (hot W0 spot*) ) | Search modes - Find all my search terms | Interface - EBSCOhost Research Databases  Search Screen - Advanced Search  Database - CINAHL Plus with Full Text | 3,292 |
| S24 | TI ( ((avoid* or avert* or circumvent* or decreas* or declin* or deter* or discourag* or dissuad* or lessen* or limit* or lower* or minimis* or minimiz* or prevent* or reduc*) N3 (hyperuse* or hyperusing or (hyper W0 use*) or "hyper-using")) ) OR AB ( ((avoid* or avert* or circumvent* or decreas* or declin* or deter* or discourag* or dissuad* or lessen* or limit* or lower* or minimis* or minimiz* or prevent* or reduc*) N3 (hyperuse* or hyperusing or (hyper W0 use*) or "hyper-using")) ) | Search modes - Find all my search terms | Interface - EBSCOhost Research Databases  Search Screen - Advanced Search  Database - CINAHL Plus with Full Text | 0 |
| S23 | TI ( ((avoid* or avert* or circumvent* or decreas* or declin* or deter* or discourag* or dissuad* or lessen* or limit* or lower* or minimis* or minimiz* or prevent* or reduc*) N3 (superuse or superused or superuses or superusing or superuser# or "super-use" or "super-used" or "super-uses" or "super-using" or "super-user" or "super-users" or superutilis* or (super W0 utilis*) or superutiliz* or (super W0 utiliz*)) ) OR AB ( ((avoid* or avert* or circumvent* or decreas* or declin* or deter* or discourag* or dissuad* or lessen* or limit* or lower* or minimis* or minimiz* or prevent* or reduc*) N3 (superuse or superused or superuses or superusing or superuser# or "super-use" or "super-used" or "super-uses" or "super-using" or "super-user" or "super-users" or superutilis* or (super W0 utilis*) or superutiliz* or (super W0 utiliz*)) ) | Search modes - Find all my search terms | Interface - EBSCOhost Research Databases  Search Screen - Advanced Search  Database - CINAHL Plus with Full Text | 14 |
| S22 | TI ( ((avoid* or avert* or circumvent* or decreas* or declin* or deter* or discourag* or dissuad* or lessen* or limit* or lower* or minimis* or minimiz* or prevent* or reduc*) N3 (overuse or overused or overuses or overusing or overuser# or "over-use" or "over-used" or "over-uses" or "over-using" or "over-user" or "over-users" or overutilis* or (over W0 utilis*) or overutiliz* or (over W0 utiliz*)) ) OR AB ( ((avoid* or avert* or circumvent* or decreas* or declin* or deter* or discourag* or dissuad* or lessen* or limit* or lower* or minimis* or minimiz* or prevent* or reduc*) N3 (overuse or overused or overuses or overusing or overuser# or "over-use" or "over-used" or "over-uses" or "over-using" or "over-user" or "over-users" or overutilis* or (over W0 utilis*) or overutiliz* or (over W0 utiliz*)) ) | Search modes - Find all my search terms | Interface - EBSCOhost Research Databases  Search Screen - Advanced Search  Database - CINAHL Plus with Full Text | 1,136 |
| S21 | TI ( ((avoid* or avert* or circumvent* or decreas* or declin* or deter* or discourag* or dissuad* or lessen* or limit* or lower* or minimis* or minimiz* or prevent* or reduc*) N3 (misuse or misused or misuses or misusing or misuser# or "mis-use" or "mis-used" or "mis-uses" or "mis-using" or "mis-user" or "mis-users")) ) OR AB ( ((avoid* or avert* or circumvent* or decreas* or declin* or deter* or discourag* or dissuad* or lessen* or limit* or lower* or minimis* or minimiz* or prevent* or reduc*) N3 (misuse or misused or misuses or misusing or misuser# or "mis-use" or "mis-used" or "mis-uses" or "mis-using" or "mis-user" or "mis-users")) ) | Search modes - Find all my search terms | Interface - EBSCOhost Research Databases  Search Screen - Advanced Search  Database - CINAHL Plus with Full Text | 1,254 |
| S20 | TI ( ((avoid* or avert* or circumvent* or decreas* or declin* or deter* or discourag* or dissuad* or lessen* or limit* or lower* or minimis* or minimiz* or prevent* or reduc*) N5 ("use" or used or user or users or uses or using or utilis* or utiliz*) N3 (visit* or attend*)) ) OR AB ( ((avoid* or avert* or circumvent* or decreas* or declin* or deter* or discourag* or dissuad* or lessen* or limit* or lower* or minimis* or minimiz* or prevent* or reduc*) N5 ("use" or used or user or users or uses or using or utilis* or utiliz*) N3 (visit* or attend*)) ) | Search modes - Find all my search terms | Interface - EBSCOhost Research Databases  Search Screen - Advanced Search  Database - CINAHL Plus with Full Text | 978 |
| S19 | TI ( ((avoid* or avert* or circumvent* or decreas* or declin* or deter* or discourag* or dissuad* or lessen* or limit* or lower* or minimis* or minimiz* or prevent* or reduc*) N3 (("ED" N3 attend*) or ("EDs" N3 attend*) or ("ED" N3 visit*) or ("EDs" N3 visit*) or (emergency department* N3 attend*) or (emergency department* N3 visit*))) ) OR AB ( ((avoid* or avert* or circumvent* or decreas* or declin* or deter* or discourag* or dissuad* or lessen* or limit* or lower* or minimis* or minimiz* or prevent* or reduc*) N3 (("ED" N3 attend*) or ("EDs" N3 attend*) or ("ED" N3 visit*) or ("EDs" N3 visit*) or (emergency department* N3 attend*) or (emergency department* N3 visit*))) ) | Search modes - Find all my search terms | Interface - EBSCOhost Research Databases  Search Screen - Advanced Search  Database - CINAHL Plus with Full Text | 2,161 |
| S18 | TI ( (((before or inside or "less than" or "not greater than" or "prior to" or under or within) N3 ("30" or thirty or one month or "1 month" or "the month" or four weeks or "4 weeks") N3 (("ED" N3 re-visit*) or ("EDs" N3 re-visit*))) or (emergency department* N3 re-visit*)) ) OR AB ( (((before or inside or "less than" or "not greater than" or "prior to" or under or within) N3 ("30" or thirty or one month or "1 month" or "the month" or four weeks or "4 weeks") N3 (("ED" N3 re-visit*) or ("EDs" N3 re-visit*))) or (emergency department* N3 re-visit*)) ) | Search modes - Find all my search terms | Interface - EBSCOhost Research Databases  Search Screen - Advanced Search  Database - CINAHL Plus with Full Text | 3 |
| S17 | TI ( (((before or inside or "less than" or "not greater than" or "prior to" or under or within) N3 ("30" or thirty or one month or "1 month" or "the month" or four weeks or "4 weeks") N3 (("ED" N3 revisit*) or ("EDs" N3 revisit*))) or (emergency department* N3 revisit*)) ) OR AB ( (((before or inside or "less than" or "not greater than" or "prior to" or under or within) N3 ("30" or thirty or one month or "1 month" or "the month" or four weeks or "4 weeks") N3 (("ED" N3 revisit*) or ("EDs" N3 revisit*))) or (emergency department* N3 revisit*)) ) | Search modes - Find all my search terms | Interface - EBSCOhost Research Databases  Search Screen - Advanced Search  Database - CINAHL Plus with Full Text | 188 |
| S16 | TI ( (((before or inside or "less than" or "not greater than" or "prior to" or under or within) N3 ("30" or thirty or one month or "1 month" or "the month" or four weeks or "4 weeks") N3 (("ED" N3 return*) or ("EDs" N3 return*))) or (emergency department* N3 return)) ) OR AB ( (((before or inside or "less than" or "not greater than" or "prior to" or under or within) N3 ("30" or thirty or one month or "1 month" or "the month" or four weeks or "4 weeks") N3 (("ED" N3 return*) or ("EDs" N3 return*))) or (emergency department* N3 return)) ) | Search modes - Find all my search terms | Interface - EBSCOhost Research Databases  Search Screen - Advanced Search  Database - CINAHL Plus with Full Text | 352 |
| S15 | TI ( (((avoid* or avert* or circumvent* or decreas* or declin* or deter* or discourag* or dissuad* or lessen* or limit* or lower* or minimis* or minimiz* or prevent* or reduc*) N3 (("ED" N3 return*) or ("EDs" N3 return*))) or (emergency department* N3 return)) ) OR AB ( (((avoid* or avert* or circumvent* or decreas* or declin* or deter* or discourag* or dissuad* or lessen* or limit* or lower* or minimis* or minimiz* or prevent* or reduc*) N3 (("ED" N3 return*) or ("EDs" N3 return*))) or (emergency department* N3 return)) ) | Search modes - Find all my search terms | Interface - EBSCOhost Research Databases  Search Screen - Advanced Search  Database - CINAHL Plus with Full Text | 341 |
| S14 | TI ( (avoid* or avert* or circumvent* or decreas* or declin* or deter* or discourag* or dissuad* or lessen* or limit* or lower* or minimis* or minimiz* or prevent* or reduc*) N3 ((repeat* N3 attend*) or (repeat* N3 visit*) or (return* N3 attend*) or (return* N3 visit*) or (frequen* N3 attend*) or (frequen* N3 visit*) or (unplanned N3 attend*) or (unplanned N3 return*) or (unplanned N3 visit*) or (unscheduled N3 attend*) or (unscheduled N3 return*) or (unscheduled N3 visit*) or (unanticipated N3 attend*) or (unanticipated N3 return*) or (unanticipated N3 visit*) or (unforeseen N3 attend*) or (unforeseen N3 return*) or (unforeseen N3 visit*) or revisit* or re-visit*) ) OR AB ( (avoid* or avert* or circumvent* or decreas* or declin* or deter* or discourag* or dissuad* or lessen* or limit* or lower* or minimis* or minimiz* or prevent* or reduc*) N3 ((repeat* N3 attend*) or (repeat* N3 visit*) or (return* N3 attend*) or (return* N3 visit*) or (frequen* N3 attend*) or (frequen* N3 visit*) or (unplanned N3 attend*) or (unplanned N3 return*) or (unplanned N3 visit*) or (unscheduled N3 attend*) or (unscheduled N3 return*) or (unscheduled N3 visit*) or (unanticipated N3 attend*) or (unanticipated N3 return*) or (unanticipated N3 visit*) or (unforeseen N3 attend*) or (unforeseen N3 return*) or (unforeseen N3 visit*) or revisit* or re-visit*) ) | Search modes - Find all my search terms | Interface - EBSCOhost Research Databases  Search Screen - Advanced Search  Database - CINAHL Plus with Full Text | 959 |
| S13 | (MM "Readmission") | Search modes - Find all my search terms | Interface - EBSCOhost Research Databases  Search Screen - Advanced Search  Database - CINAHL Plus with Full Text | 8,076 |
| S12 | S1 OR S2 OR S3 OR S4 OR S5 OR S6 OR S7 OR S8 OR S9 OR S10 OR S11 | Search modes - Find all my search terms | Interface - EBSCOhost Research Databases  Search Screen - Advanced Search  Database - CINAHL Plus with Full Text | 141,434 |
| S11 | TI "ED" N5 emergenc* OR AB "ED" N5 emergenc* | Search modes - Find all my search terms | Interface - EBSCOhost Research Databases  Search Screen - Advanced Search  Database - CINAHL Plus with Full Text | 19,916 |
| S10 | TI ( (urgent or trauma) N3 (care or healthcare or "health care") ) OR AB ( (urgent or trauma) N3 (care or healthcare or "health care") ) | Search modes - Find all my search terms | Interface - EBSCOhost Research Databases  Search Screen - Advanced Search  Database - CINAHL Plus with Full Text | 9,479 |
| S9 | TI ( trauma N0 (centre or centres or center or centers) ) OR AB ( trauma N0 (centre or centres or center or centers) ) | Search modes - Find all my search terms | Interface - EBSCOhost Research Databases  Search Screen - Advanced Search  Database - CINAHL Plus with Full Text | 10,421 |
| S8 | TI ( hospital* N3 ("emergency service" or "emergency services") ) OR AB ( hospital* N3 ("emergency service" or "emergency services") ) | Search modes - Find all my search terms | Interface - EBSCOhost Research Databases  Search Screen - Advanced Search  Database - CINAHL Plus with Full Text | 421 |
| S7 | TI ( emergency N0 (healthcare or "health care" or medical) N0 service# ) OR AB ( emergency N0 (healthcare or "health care" or medical) N0 service# ) | Search modes - Find all my search terms | Interface - EBSCOhost Research Databases  Search Screen - Advanced Search  Database - CINAHL Plus with Full Text | 7,086 |
| S6 | TI ( "A and E" N2 (center or centers or centre or centres or department# or dept or depts or room or rooms or unit or units or ward or wards) ) OR AB ( "A and E" N2 (center or centers or centre or centres or department# or dept or depts or room or rooms or unit or units or ward or wards) ) | Search modes - Find all my search terms | Interface - EBSCOhost Research Databases  Search Screen - Advanced Search  Database - CINAHL Plus with Full Text | Display |
| S5 | TI ( accident N2 (center or centers or centre or centres or department# or dept or depts or room or rooms or unit or units or ward or wards) ) OR AB ( accident N2 (center or centers or centre or centres or department# or dept or depts or room or rooms or unit or units or ward or wards) ) | Search modes - Find all my search terms | Interface - EBSCOhost Research Databases  Search Screen - Advanced Search  Database - CINAHL Plus with Full Text | Display |
| S4 | TI ( emergency N2 (center or centers or centre or centres or department# or dept or depts or room or rooms or unit or units or ward or wards) ) OR AB ( emergency N2 (center or centers or centre or centres or department# or dept or depts or room or rooms or unit or units or ward or wards) ) | Search modes - Find all my search terms | Interface - EBSCOhost Research Databases  Search Screen - Advanced Search  Database - CINAHL Plus with Full Text | Display |
| S3 | TI "emergency medicine" OR AB "emergency medicine" | Search modes - Find all my search terms | Interface - EBSCOhost Research Databases  Search Screen - Advanced Search  Database - CINAHL Plus with Full Text | Display |
| S2 | (MH "Emergency Medicine") | Search modes - Find all my search terms | Interface - EBSCOhost Research Databases  Search Screen - Advanced Search  Database - CINAHL Plus with Full Text | Display |
| S1 | (MH "Emergency Service+") | Search modes - Find all my search terms | Interface - EBSCOhost Research Databases  Search Screen - Advanced Search  Database - CINAHL Plus with Full Text | Display |

2023 Jan 19

Cochrane Library

ID Search Hits

#1 [mh "Emergency Service, Hospital"] 2812

#2 [mh "Emergency Medicine"] 300

#3 "emergency medicine":ti,ab,kw 2154

#4 (emergency NEAR/2 (center or centers or centre or centres or department? or dept or depts or room or rooms or unit or units or ward or wards)):ti,ab,kw 15809

#5 (accident NEAR/2 (center or centers or centre or centres or department? or dept or depts or room or rooms or unit or units or ward or wards)):ti,ab,kw 46

#6 ("A and E" NEAR/2 (center or centers or centre or centres or department? or dept or depts or room or rooms or unit or units or ward or wards)):ti,ab,kw 1

#7 (emergency NEXT (healthcare or "health care" or medical) NEXT service*):ti,ab,kw 1623

#8 (hospital* NEAR/3 ("emergency service" or "emergency services")):ti,ab,kw 2748

#9 (trauma NEXT (centre or centres or center or centers)):ti,ab,kw 1304

#10 ((urgent or trauma) NEAR/3 (care or healthcare or "health care")):ti,ab,kw 1067

#11 ("ED" NEAR/5 emergenc*):ti,ab,kw 3832

#12 ^104-#11^ 20391

#13 [mh "Patient Readmission" [mj]] 248

#14 ((avoid* or avert* or circumvent* or decreas* or declin* or deter* or discourag* or dissuad* or lessen* or limit* or lower* or minimis* or minimiz* or prevent* or reduc*) NEAR/3 ((repeat* NEAR/3 attend*) or (repeat* NEAR/3 visit*) or (return* NEAR/3 attend*) or (return* NEAR/3 visit*) or (frequen* NEAR/3 attend*) or (frequen* NEAR/3 visit*) or (unplanned NEAR/3 attend*) or (unplanned NEAR/3 return*) or (unplanned NEAR/3 visit*) or (unscheduled NEAR/3 attend*) or (unscheduled NEAR/3 return*) or (unscheduled NEAR/3 visit*) or (unanticipated NEAR/3 attend*) or (unanticipated NEAR/3 return*) or (unanticipated NEAR/3 visit*) or (unforeseen NEAR/3 attend*) or (unforeseen NEAR/3 return*) or (unforeseen NEAR/3 visit*) or revisit* or re-visit*)):ti,ab,kw 270

#15 (((avoid* or avert* or circumvent* or decreas* or declin* or deter* or discourag* or dissuad* or lessen* or limit* or lower* or minimis* or minimiz* or prevent* or reduc*) NEAR/3 ((ED NEAR/3 return*) or (EDs NEAR/3 return*))) or (emergency department* NEAR/3 return)):ti,ab,kw 36

#16 (((before or inside or "less than" or "not greater than" or "prior to" or under or within) NEAR/3 ("30" or thirty or one month or "1 month" or "the month" or four weeks or "4 weeks") NEAR/3 ((ED NEAR/3 return*) or (EDs NEAR/3 return*))) or (emergency department* NEAR/3 return)):ti,ab,kw 35

#17 (((before or inside or "less than" or "not greater than" or "prior to" or under or within) NEAR/3 ("30" or thirty or one month or "1 month" or "the month" or four weeks or "4 weeks") NEAR/3 ((ED NEAR/3 revisit*) or (EDs NEAR/3 revisit*))) or (emergency department* NEAR/3 revisit*)):ti,ab,kw 30

#18 (((before or inside or "less than" or "not greater than" or "prior to" or under or within) NEAR/3 ("30" or thirty or one month or "1 month" or "the month" or four weeks or "4 weeks") NEAR/3 ((ED NEAR/3 re-visit*) or (EDs NEAR/3 re-visit*))) or (emergency department* NEAR/3 re-visit*)):ti,ab,kw 1

#19 ((avoid* or avert* or circumvent* or decreas* or declin* or deter* or discourag* or dissuad* or lessen* or limit* or lower* or minimis* or minimiz* or prevent* or reduc*) NEAR/3 ((ED NEAR/3 attend*) or (EDs NEAR/3 attend*) or (ED NEAR/3 visit*) or (EDs NEAR/3 visit*) or (emergency department* NEAR/3 attend*) or (emergency department* NEAR/3 visit*))):ti,ab,kw 1382

#20 ((avoid* or avert* or circumvent* or decreas* or declin* or deter* or discourag* or dissuad* or lessen* or limit* or lower* or minimis* or minimiz* or prevent* or reduc*) NEAR/5 ("use" or used or user or users or uses or using or utilis* or utiliz*) NEAR/3 (visit* or attend*)):ti,ab,kw 275

#21 ((avoid* or avert* or circumvent* or decreas* or declin* or deter* or discourag* or dissuad* or lessen* or limit* or lower* or minimis* or minimiz* or prevent* or reduc*) NEAR/3 (misuse or misused or misuses or misusing or misuser* or "mis-use" or "mis-used" or "mis-uses" or "mis-using" or mis-user*)):ti,ab,kw 341

#22 ((avoid* or avert* or circumvent* or decreas* or declin* or deter* or discourag* or dissuad* or lessen* or limit* or lower* or minimis* or minimiz* or prevent* or reduc*) NEAR/3 (overuse or overused or overuses or overusing or overuser* or "over-use" or "over-used" or "over-uses" or "over-using" or "over-user" or "over-users" or overutilis* or over-utilis* or overutiliz* or over-utiliz*)):ti,ab,kw 248

#23 ((avoid* or avert* or circumvent* or decreas* or declin* or deter* or discourag* or dissuad* or lessen* or limit* or lower* or minimis* or minimiz* or prevent* or reduc*) NEAR/3 (superuse or superused or superuses or superusing or superuser* or "super-use" or "super-used" or "super-uses" or "super-using" or "super-user" or "super-users" or superutilis* or super-utilis* or superutiliz* or super-utiliz*)):ti,ab,kw 0

#24 ((avoid* or avert* or circumvent* or decreas* or declin* or deter* or discourag* or dissuad* or lessen* or limit* or lower* or minimis* or minimiz* or prevent* or reduc*) NEAR/3 (hyperuse* or hyperusing or hyper-use* or hyper-using)):ti,ab,kw 0

#25 (hotspot* or (hot NEXT spot*)):ti,ab,kw 545

#26 (((((return* NEAR/2 visit*) or (return* NEAR/2 attend*) or (repeat* NEAR/2 visit*) or (repeat* NEAR/2 attend*) or revisit* or re-visit*) and (post or after or following* or home or initial* or newly or recent*)) NEAR/3 discharg*) or postdischarg* or "post-ED" or "post-hospitalisation" or "post-hospitalization"):ti,ab,kw 16560

#27 ^105-#26^ 19458

#28 #12 AND #27 with Cochrane Library publication date Between Jan 2013 and Jan 2023, in Cochrane Reviews, Cochrane Protocols, Trials 2181

Reviews – 20

Trials - 2161
